# Computational Integration of Homolog and Pathway Gene Module Expression Reveals General Stemness Signatures

**DOI:** 10.1371/journal.pone.0018968

**Published:** 2011-04-29

**Authors:** Martina Koeva, E. Camilla Forsberg, Joshua M. Stuart

**Affiliations:** Biomolecular Engineering, University of California Santa Cruz, Santa Cruz, California, United States of America; Centre for Genomic Regulation, Spain

## Abstract

The stemness hypothesis states that all stem cells use common mechanisms to regulate self-renewal and multi-lineage potential. However, gene expression meta-analyses at the single gene level have failed to identify a significant number of genes selectively expressed by a broad range of stem cell types. We hypothesized that stemness may be regulated by modules of homologs. While the expression of any single gene within a module may vary from one stem cell type to the next, it is possible that the expression of the module as a whole is required so that the expression of different, yet functionally-synonymous, homologs is needed in different stem cells. Thus, we developed a computational method to test for stem cell-specific gene expression patterns from a comprehensive collection of 49 murine datasets covering 12 different stem cell types. We identified 40 individual genes and 224 stemness modules with reproducible and specific up-regulation across multiple stem cell types. The stemness modules included families regulating chromatin remodeling, DNA repair, and Wnt signaling. Strikingly, the majority of modules represent evolutionarily related homologs. Moreover, a score based on the discovered modules could accurately distinguish stem cell-like populations from other cell types in both normal and cancer tissues. This scoring system revealed that both mouse and human metastatic populations exhibit higher stemness indices than non-metastatic populations, providing further evidence for a stem cell-driven component underlying the transformation to metastatic disease.

## Introduction

Stem cells are defined by their ability to both self-renew and differentiate into mature cells. In addition to their functions in development, stem cells play key roles in degenerative disease, aging, and oncogenesis. Cancer stem cells may promote tumor heterogeneity and metastasis. Identifying genes regulating stem cell properties will greatly improve our understanding of the molecular mechanisms regulating stem cell functions, our ability to manipulate stem cell fate, and the roles of stem cells in cancer.

**Figure 1:**
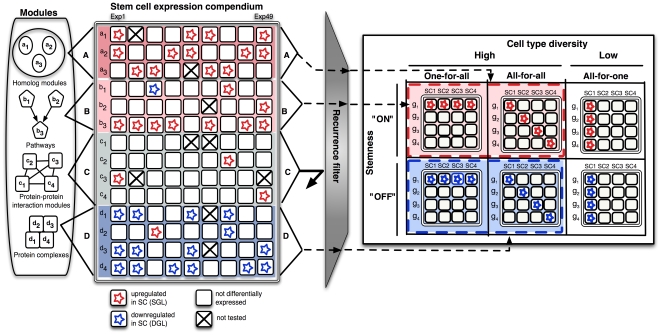
Overview of the stemness meta-analysis pipeline (S-MAP). Gene modules were derived from several sources: A, homologs; B, pathways; C, protein-protein interaction groups; D, protein complexes (rows labeled a_1_, a_2_, …, b_1_, b_2_, … represent individual genes in each module). Expression from 49 studies (columns in left panel) was collected containing genes up-regulated (red) or down-regulated (blue) in stem cells. Modules with genes highly recurrent in stem cells were classified as “stemness-on” (modules A and B; red region) and those with recurrent in differentiated cells as “stemness-off” (module D; blue region) if the pattern was specific and found to extend across many cell types (columns in right panel labeled SC1, SC2, …) based on cell-diversity.

The stemness hypothesis has been debated and so far no conclusive evidence for a set of genes expressed in all stem cells (“one-for-all” pattern, Figure 1) has been reported [1], [2], [3], [4]. Fortunel *et al.* [1] compared up-regulated genes from hematopoietic, neural, retinal and embryonic stem cells in mouse and uncovered only a single shared gene, integrin α6 (Itga6), with previous stem cell expression experiments. Subsequent studies in human and mouse found little overlap in the genes uncovered by these studies [2]. More recently, pathway-level analyses have advanced our understanding of stem cell mechanisms. For example, Muller *et al.* [5] constructed a “PluriNet,” of interacting genes with a focus to identify mechanisms that differ between different stem cell types. Wong *et al.* (2008) [6] identified sets of genes in a common pathway or physically interacting proteins as significantly co-regulated in embryonic stem cells.

We hypothesized that homologs, genes in an organism that share a recent common ancestral gene, may play compensatory or overlapping roles in stem cells. Single gene- and pathway-level analyses overlook the possible complementary activity of homologs. Paralogs may diverge in function to evolve roles in parallel pathways that control common processes but are employed in different contexts such as distinct tissues. To test for coordinate regulation of gene homologs or modules across different stem cell types, we developed a pattern recognition algorithm capable of combining the results of any number of experiments to identify significantly and recurrently up- or down-regulated genes and gene modules in stem cells.

## Results

### Derivation of modules and datasets

We compiled gene modules from either homologs or functionally related sets of genes that co-participate in pathways, protein-protein interactions, and protein complexes (Figure 1). Homolog modules were determined using a genome-wide BLAST analysis [7], which assigned 24,403 protein-coding genes in the mouse genome to 4,657 mutually exclusive groups and 5,251 *biological singletons* predicted to lack a close homolog in mouse (Figure S1a). The groups had an 88% correspondence to HomoloGene [8] clusters (Figure S1b). Non-redundant functional modules were collected from a wide range of sources, including Gene Ontology [9], Kyoto Encyclopedia of Genes and Genomes [10] and BioCarta [11] pathways (376 modules), experimentally derived mouse protein complexes (90 modules), and mouse and human protein-protein interaction data (145 modules) (Dataset S1).

To obtain a global overview of stem cell expression patterns, we assembled a compendium of data from 30 different studies assaying gene expression of 49 stem cell populations representing twelve different types of stem cells including hematopoietic, retinal, neural, embryonic, and intestinal (Figure 1; Table 1). From each of the 49 datasets we collected genes up-regulated in stem cells into a stem cell gene list (SGL) and, where available, a corresponding set of genes up-regulated in differentiated cells into a differentiated gene list (DGL) (Table S1 and Dataset S2). The use of gene lists facilitated the straightforward integration of results from the variety of experimental test platforms, which has proven effective for meta-analysis compared to alternative approaches [12].

**Table 1:** Stem cell types analyzed in this study.

		No. of	No. of	No. of
Stem Cell Type	Abbrev	Studies	SGLs	DGLs
Hematopoietic	HSC	8	12	17
Retinal	RPC	1	1	1
Neural	NSC	7	7	7
Embryonic	ESC	8	14	14
Mesenchymal	MSC	2	2	1
Gastric	GEP	2	2	1
Intestinal	InSC	3	3	2
Liver	LiSC	1	1	1
Breast	MaSC	1	1	1
Hair follicle	HBSC	2	2	1
Spermatogonial	SSC	3	3	3
Trophoblast	TSC	1	1	1

### Computational framework to identify stemness

We developed a Stemness Meta-Analysis Pipeline (S-MAP) to test for modules and individual genes coordinately up-regulated (stemness-on) or down-regulated (stemness-off) in stem cells from the SGLs and DGLs (Figure 1). S-MAP tests for significant stemness-associated expression of a particular gene or module by computing three scores – *recurrence*, *specificity*, and *diversity* (Figure 1). The recurrence score measures the overall amount of up- or down-regulation across all experiments that controls for redundancy across datasets (Table S2). An empirical false-discovery rate (FDR) for the recurrence score was determined by simulating random modules of each size and defining scores with associated FDR<5% as high, FDR>95% as low, and intermediate FDR as a moderate (Figure S2a). The majority of the discovered modules could also be identified using a sub-compendium containing the results from either the cultured or the non-cultured subset of stem cells from the main compendium (Figure S2b) suggesting that the observed levels of coordinated expression were not due to the cultured conditions of a subset of the cells.

For all highly recurrent modules S-MAP computes an information-theoretic *cell-diversity* score from the proportions of up-regulated events. High scores reflect upregulation in a wide range of the twelve stem cells and were defined as those with FDRs less than 5% determined by random simulation (Figure S2c–d). Modules with high cell-diversity had a high fraction of up-regulated genes across multiple stem cell types. In addition, recurrent modules were classified by *gene-diversity* to measure the extent to which multiple genes within each module were up-regulated. Based on these two diversity measures, modules were classified into six possible idealized patterns to enable further investigation of distinct module classes (see Methods). Here we discuss the *all-for-all* and *all-for-one* classes because these modules exhibit the clearest examples of stemness-related expression. The *all-for-all* class contains modules in which many gene members were up-regulated in most stem cell types whereas the *one-for-all* class contain modules in which a single gene is predominantly up-regulated across the stem cells (Figure 1). In contrast, lineage-specific modules, not discussed further here, were classified into the *all-for-one* class and contain modules in which member genes were found to be up-regulated in a limited set of stem cell types.

To pinpoint modules specifically associated with stemness, all-for-all modules were tested for expression exclusive to stem cells. We defined “stemness-on” modules as those modules classified as either *all-for-all* or *one-for-all* when the SGL compendium was used as the input data and were not classified by S-MAP when using the DGL compendium. We discuss those genes and modules with specific over-expression in either stem or differentiated cells. However, important modules may exhibit expression in both stem and differentiated cells. A full list of classified modules is available as Supporting Information (Dataset S1).

### Identification of stemness modules

**Figure 2:**
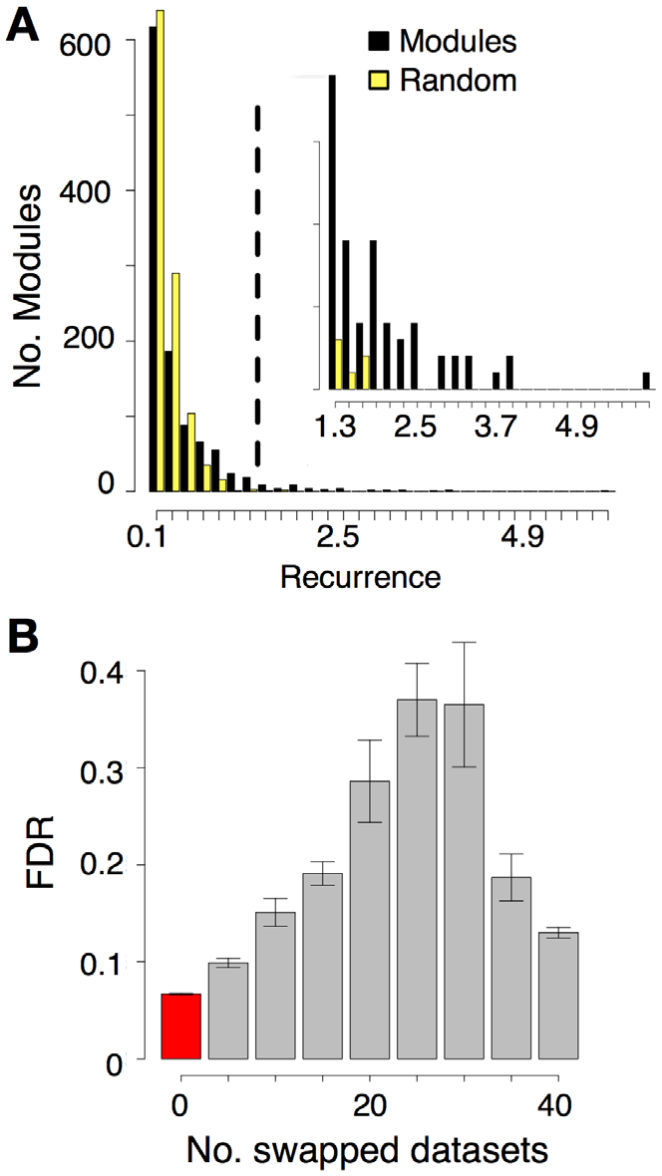
Gene modules exhibit significantly recurrent expression across diverse stem cells. (A) Recurrence score distribution compared to a background control. All modules of size 3 (1098 modules: 1091 homolog modules and 7 functional modules; black), compared to randomized families of size 3 based on 1000 random permutations of the original data (yellow). Similar results were obtained for modules of other sizes (Figure S2e–g). (B) Swap experiment. An increasing number of SGLs were replaced by their corresponding DGL and used to calculate recurrence for all modules. For several swap proportions (x-axis) the average FDR (*y-*axis) of the computed recurrences was plotted (gray bars) and compared to the distribution obtained without swapping (red bars). Error bars depict one standard error.

S-MAP identified 350 non-redundant up-regulated modules with reproducibly higher expression in stem cells compared to their matched differentiated cells across the compendium (<5% recurrence FDR). These included 266 homolog modules (2.7%) and 94 functional modules (15.3%) both of which exhibited a significant shift to higher scores compared to control modules made up of sets of randomly grouped genes (*t*-statistic = 7.2669; *P*<3.498e-13; Figure 2A; Figure S2e–g). Examples of several of the stemness-on modules are listed in Table 2 and include genes recognizable for their roles in stemness and cancer including Myb-, Myc-, P53-, and Tcf-related families. Similarly, using DGLs, S-MAP identified 213 homolog and 44 functional modules with recurrent down-regulation. Thus, S-MAP was able to identify a significant number of additional modules untested by previous attempts by including those defined by homology.

**Table 2:** Selected stemness-on homolog modules with high recurrence and specificity.

Stemness		
modules	Size	Gene Members
Myb	3	a-myb,b-myb,c-myb
Tcf/Lef	4	Tcf3, Tcf7, Lef1
Myc	6	c-myc,N-Myc,L-Myc,s-myc
P53	3	p53, p63, p73
Cip/Kip	3	p21, p27, p57
Sfrp	3	Sfrp1, Sfrp2, Sfrp5
Pbx	5	Pbx1-4
Smarc/Chd	>10	Chd1-9,Smarca1-5
Aurora	5	Aurka,Aurkb,Aurkc,Plk4,Ulk3
Integrin alpha	10	Itga2b-9,Itgav,Gpld1
Mcm	9	Mcm2-9
Nme	4	Nme1-4

As a negative control, we tested whether the observed levels of recurrence exceeded what would be expected from a diverse collection of unrelated cells. To do this, we performed a “swap” experiment in which a proportion of SGLs were swapped with an increasing number of their DGL counterparts. The FDR increased when as little as five SGLs were replaced by DGLs and continued to increase with additional swaps (10, 15, 20 and 25 swaps) such that datasets containing about equal numbers of SGLs and DGLs (25 swaps) yielded the highest FDR. Once the majority of the data contained DGLs, the FDRs decreased, suggesting that the differentiated cells also share overlapping expression patterns. Importantly, however, the lowest FDR (6.6%±0.0006) was achieved with the original SGL dataset (red bar, Figure 2B) revealing that stem cells exhibit a higher level of coordinated expression than unrelated cells.

**Figure 3:**
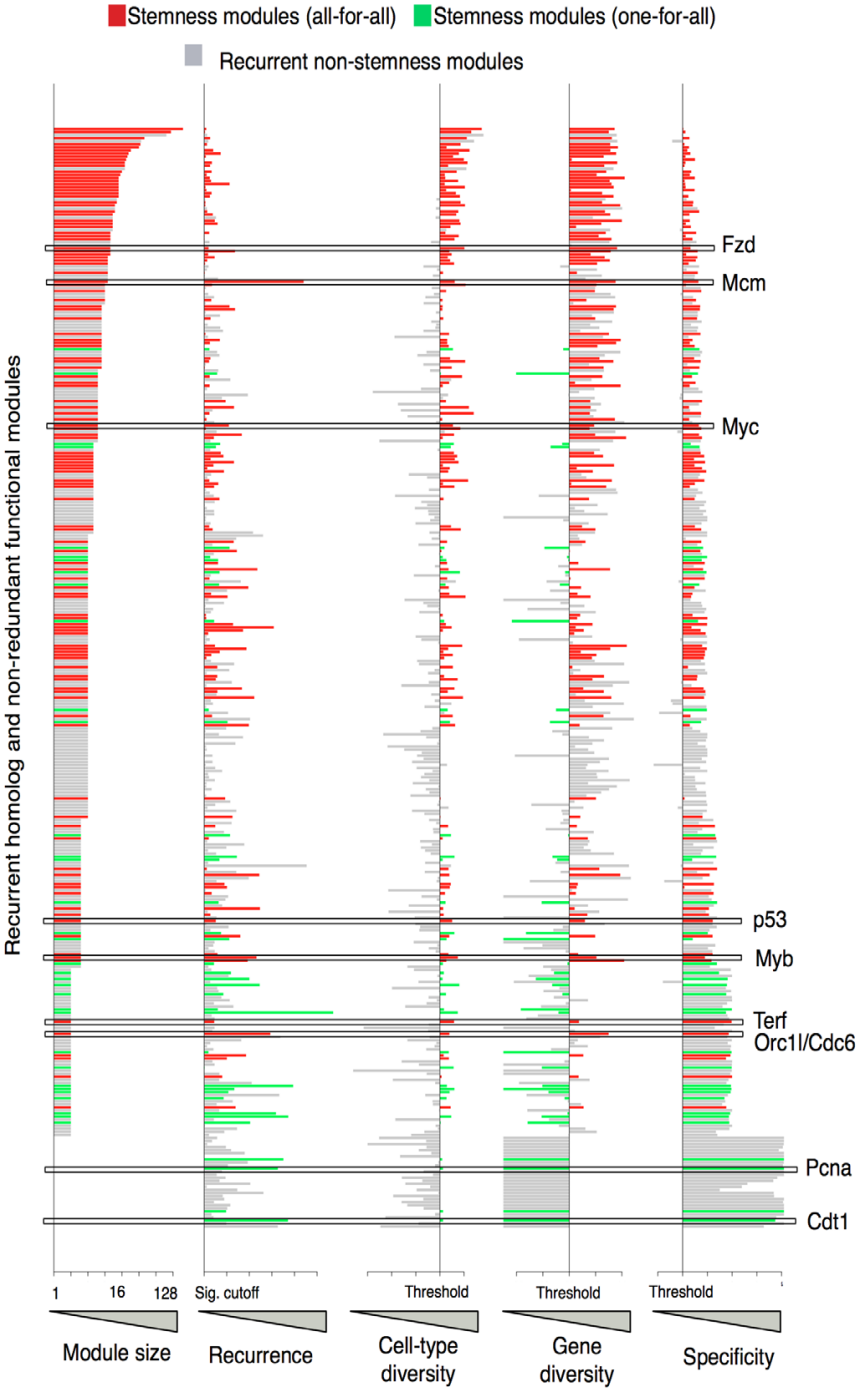
Multidimensional pattern classification reveals stemness-on modules. Module sizes (column 1), recurrence (column 2), cell type diversity, gene diversity and specificity (columns 3–5) are shown for all highly recurrent modules. Each line represents one module. Modules with high cell-diversity and specificity were classified as *stemness-on* (red and green). S-MAP classified modules with high gene-diversity (many genes within the module contribute to the stemness score) as *all-for-all* (red) and those with low gene-diversity (eg, only one gene within a module contributes to the stemness score) as *one-for-all* (green). Modules scoring as non-cell-diverse or non-specific were designated as non-stemness (gray). Non-recurrent modules are not displayed. Boxes and gene names highlight examples discussed in the text.

Strikingly, of the 350 modules with significant recurrence, 162 stemness-on and 62 stemness-off modules had significant cell-diversity and specificity scores (Figure 3). The stemness-on modules contained 103 homolog modules (78 all-for-all and 25 one-for-all) and 59 functional modules (46 all-for-all and 13 one-for-all) (Dataset S1). The stemness-off modules were composed of 39 homolog and 23 functional modules each representing putative programs requiring active repression for the maintenance of stemness (Dataset S1). A comparison to the results of Wong *et al.* (2008) [6] demonstrated that the homolog modules were enriched for modules of higher recurrence than the modules reported in the Stemness Module Map (Figure S3).

We also applied S-MAP to every individual gene in the mouse genome by considering each gene as a “singleton module.” Remarkably, 40 stemness-on genes were identified with significant recurrence, cell-diversity and specificity most of which have some link to functions in cell proliferation (Dataset S3). All but three of these genes were members of recurrent modules. Interestingly, the gene with the highest cell-diversity was Orc1l, a homolog to the yeast origin of replication complex recently implicated to play a role in stem cell maintenance in ESCs [13], [14]. Thus, by using a larger expression compendium, S-MAP was able to expand the number of individual genes implicated in stemness beyond the one gene, Itga6, identified by the original founder studies.

### Global map of stemness modules

**Figure 4:**
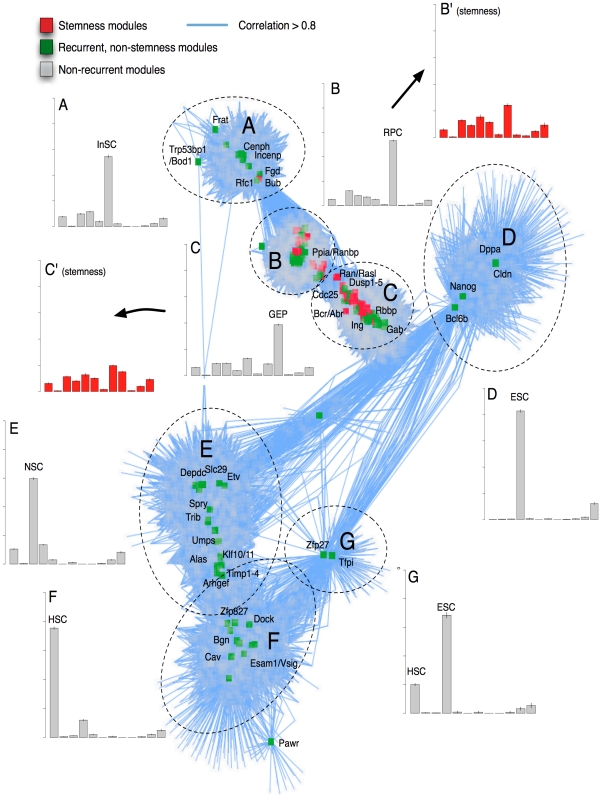
A gene expression map of modules reveals seven major components. Two modules (nodes) were connected by an edge (blue links) if their Pearson correlation exceeded 0.8 and one of the modules was recurrent (green nodes) or classified as stemness-on by S-MAP (red nodes). Non-recurrent modules connected to recurrent or stemness-on modules are also shown (grey nodes). For each component in the network (labeled A through G), the mean fraction of upregulated genes in each cell type was computed (gray bar graphs). Cell types listed on the x-axis from left to right: HSC (hematopoietic), MaSC (mammary), NSC (neural), ESC (embryonic), LiSC (liver), InSC (intestinal), MSC (mesenchymal), RPC (retinal), GEP (gastric), HBSC (hair bulge), SSC (spermatogonial), TSC (trophoblast). For the B and C components, upregulation fractions were computed for stemness-on modules only (red bar graphs).

We connected recurrent modules with similar expression patterns and plotted the resulting network using a spring-embedded layout algorithm (Figure 4). Seven major components were revealed; two contained the stemness-on modules, whereas modules with genes expressed predominantly in only one or two stem cell types characterized the remaining five components. To identify interaction networks enriched in stem cells, we applied Ingenuity Pathway Analysis for the components containing stemness-on modules (components B and C, Figure 4). The highest scoring networks included cell cycle; embryonic, tissue and development; cancer; and DNA replication and repair (Figure S4a–e). The enrichment of these networks in stem cells supports tight control of these processes across stem cell types, and suggest a link to the molecular regulation of cancer.

### Functional implications of the stemness modules

**Figure 5:**
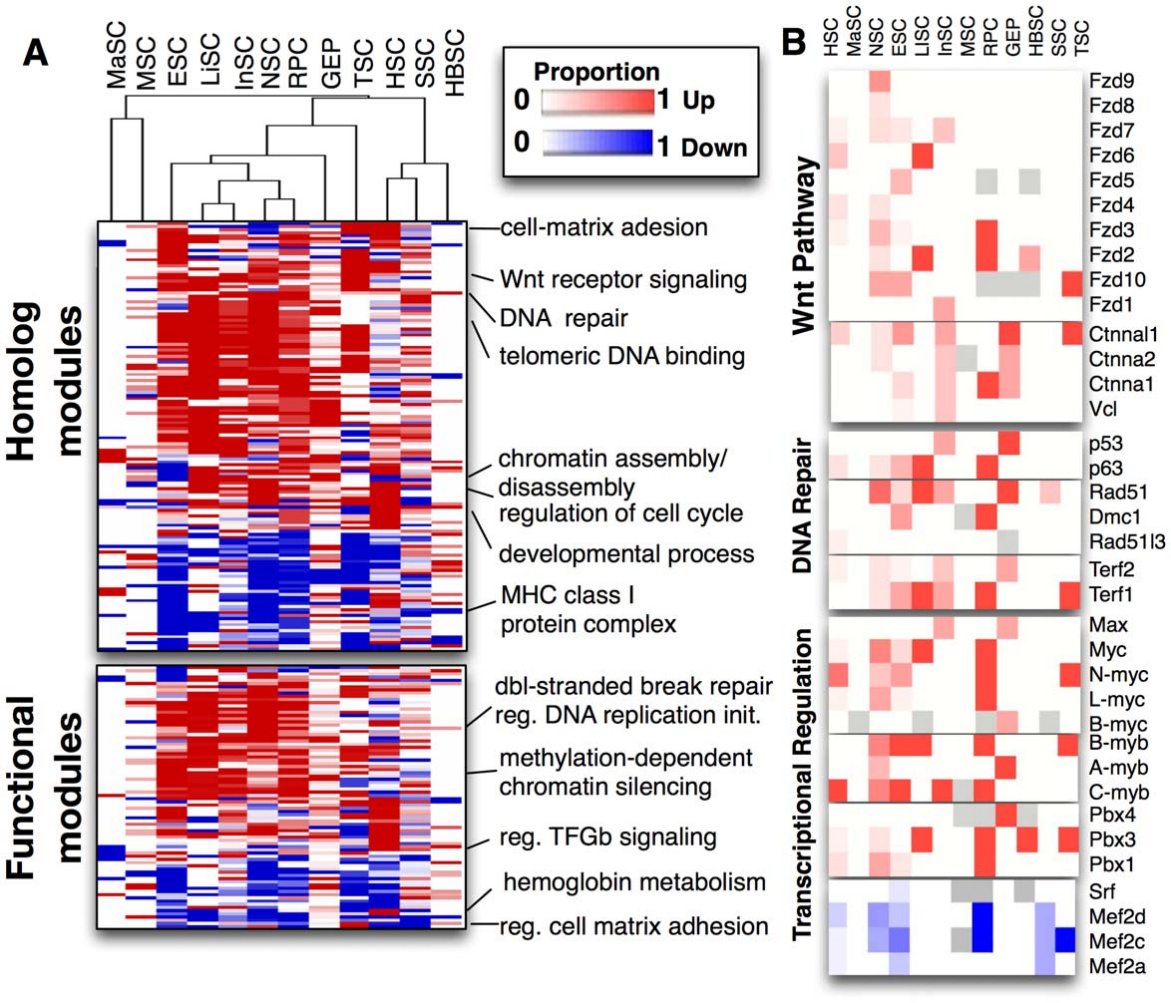
Stemness-on and stemness-off modules identified by S-MAP. **A**. Heatmap entries show the difference in the proportion of over-expressed (red) compared to under-expressed (blue) genes in each stemness-on and –off module (rows) for each type of stem cell (columns). Homolog modules are depicted above the functional modules, defined by gene functional similarity. Selected significantly enriched GO functional categories are indicated. **B**. Expression heatmaps of selected stemness-on and –off homolog modules in the Wnt, DNA repair, and transcriptional regulation categories. Event matrices for individual modules are separated by spaces. Each entry in an event matrix corresponds to the proportion of experiments in which a gene was up-regulated in a particular cell type, ranging from 0 (white; up-regulated in zero experiments) to 1 (red, up-regulated in all experiments, or blue, down-regulated in stem cells). Gray entries correspond to genes untested in any of the experiments for a specific stem cell type. Genes without any data in the compendium were excluded from the plot.

To identify functional themes, we next clustered all of the stemness-on and –off modules detected by S-MAP according to their patterns across the expression compendium (Figure 5A). Several specific processes were identified including chromatin assembly, Wnt signaling, DNA repair, and cell proliferation. Intriguingly, the integrin alpha module, including Itga6 identified by Fortunel *et al*, was among the top-scoring stemness-on homolog modules. The integrin alpha module, but no single member gene, was up-regulated in many stem cell types in addition to the ones originally investigated. Some of the most compelling all-for-all modules are discussed next; a comprehensive view is available as Supporting Information (Dataset S1).

A permissive, or poised, chromatin structure may underlie stem cell multipotency. S-MAP detected several chromatin-related modules associated with stemness including those involved in imprinting, chromatin-dependent silencing (PRC1 and PRC2 complexes), heterochromatin and the nuclear lamina, which may indicate widespread suppression of lineage-associated genes. Stemness contributors include the Chd/Smarca family, nucleosome assembly protein (Nap) like proteins; and histone variants H2afz and H2afv (one-for-all pattern; Figure S5a). Indeed, Chd1 was recently shown to be important in ESC multipotency by regulating chromatin structure [15]; other family members may fill this function in adult stem cells.

S-MAP also revealed a number of Wnt signaling modules with alternating patterns of specificity in different stem cell types (Figure 5B; Figure S5b). Wnt-related stemness modules include the secreted Frizzled-like proteins of the Sfrp family; the Frizzled receptors; a subfamily of the TCF/LEF transcriptional regulators; the Enhancer of Split/Groucho-related Tle factors; and both alpha- and delta-catenins. Whether Wnt signaling regulates stem cell maintenance or differentiation has been extensively debated, and some genes have both inhibitory and activating abilities dependent on the state of Wnt signals [16]. While functional consequences cannot be resolved by expression data alone, our analysis demonstrates that all stem cells tightly regulate the Wnt pathway at the transcriptional level. The identified S-MAP patterns provide specific candidates for functional interrogation in different stem cell types.

DNA repair is uniquely critical in stem cells as mutations accumulated in stem cells amplify in differentiated daughters. S-MAP identified many stemness modules associated with DNA repair, including the Terf, p53, and Rad families (Figure 5B; Figure S5c). Terf1 and 2 are highly stem cell-specific and are expressed in alternating patterns in different stem cells (Figure 5B). S-MAP classified the p53 family as a stemness-on module. While p53 was found to be expressed in several tissues, p63 was up-regulated in gastric and intestinal cells consistent with its known role in the development and maintenance of epithelial stem cells [17]. Likewise, Brca1 and its homolog Mcph1, the Msh family of proteins, and a Rad/Dmc module were detected as stemness modules by S-MAP (Figure S5c).

Several transcriptional regulators, including the Myb family of oncogenes, were among the highest scoring stemness-on modules (Figure 5B; Figure S5d). c-myb was enriched in hematopoietic stem cells, consistent with its known role in differentiation control, as well as neural, embryonic, intestinal and retinal stem cells. a-myb complements the expression of its partner genes by significant up-regulation in gastric stem cells, while b-myb was up-regulated in liver and trophoblast stem cells. The Pbx and Id families have also been implicated in maintaining stem cell function (Figure 5B, Figure S5d) [18], [19], [20], [21], [22]. The myc family displayed a strongly complementary stemness expression pattern. c-myc has been implicated in reprogramming of differentiated cells into pluripotent cells [23]. Not surprisingly, c-myc is the only gene of the four original iPS reprogramming factors that is represented in a stemness-on module; the other three factors (Sox2, Nanog and Oct4) are ESC-specific and not expressed by most adult stem cells (Figure 4).

Many modules representing cell proliferation or control of the cell cycle were stemness-specific. Strikingly, three Mcm proteins (Mcm2, 4 and 5) and several proteins that cooperate with Mcm’s (Cdt1, Orc1, Pcna, and Cdc6) were identified (Figure S5e). Other genes with known roles in cancer included Smoothened, a G-protein coupled receptor in the hedgehog pathway; the transcriptional activator Eya2; the histone-binding oncoprotein SET; and the mTOR inhibitor Depdc6.

S-MAP identified 62 stemness-off modules including cytokine and growth factor signaling (e.g., Pdgf and Fgf receptors; Jak signaling proteins; Src and Fyn and their homologs), actin-interacting proteins (ankyrins; gelsolin and homologs), and regulators of transcription (e.g., basic helix-loop-helix factors Bhlhb2 and 3; the Cebp family; the Mef2 family (Figure 5B)). Several of these genes are known to promote differentiation. Notably, Mef2 transcription factors may act as transcriptional on/off switches, as they preferentially interact with histone deacetylases in undifferentiated cells and with histone acetylases in differentiating cells [24], [25].

### Module-informed prediction of stemness

**Figure 6:**
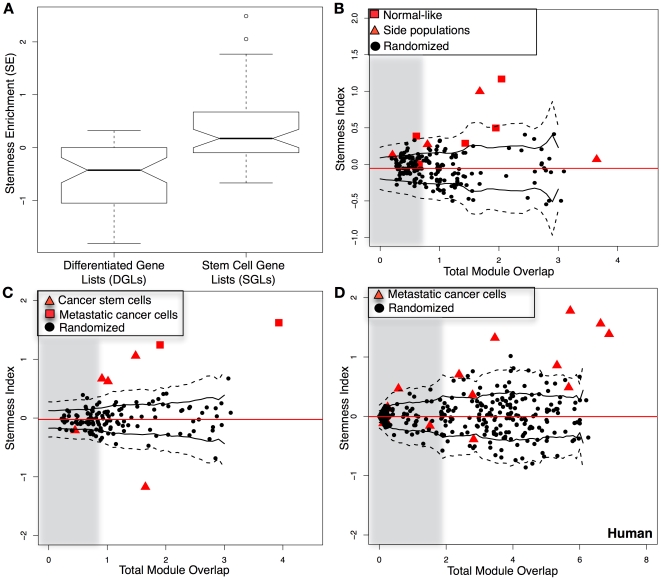
S-MAP modules predict stemness in validation data and in cancer metastases in mouse and human. (**A**) A Stemness Enrichment score (*SE*; *y*-axis) between each experimental set of up-regulated genes – for all DGLs (left boxplot) and all SGLs (right boxplot) – was measured. Positive scores indicate higher overlap with stemness-on compared to stemness-off modules. (**B–D**) A Stemness Index (*SI*) was calculated for each experiment as the difference between the Stemness Enrichment scores computed for its up- and down-regulated lists of genes (*y*-axis) and is plotted against a Total Module Overlap (*x*-axis). For each query experiment (red squares and red triangles), 20 matched random experiments were simulated to construct a null distribution for both the *SI* and Total Module Overlap (black circles). Gray regions are drawn to enclose half of the random controls. The mean *SI* from the null distribution (solid red line) is shown along with one standard deviation (black solid lines) and two standard deviations (black dashed lines) estimated using a windowing approach. Differentially expressed gene lists were scored for (**B**) mouse normal stem cells and side populations; (**C**) mouse cancer stem cells and metastatic tumors; and (**D**) human metastatic cancer cell populations.

We hypothesized that the modules recovered by S-MAP could be used to classify transcriptomes into either stemness or differentiation programs. We first computed a stemness enrichment score (*SE*) to measure the difference in the average overlap of a set of up-regulated genes to stemness-on compared to stemness-off modules (see Methods). A cross-validation test confirmed that the held-out SGLs received significantly higher SE scores than the held-out DGLs (t = 8.777; p-value  =  6.204e-16; Welch two-sample t-test; Figure 6A) and each was higher than random control modules of matching sizes (t = 4.802; *P<*2.107e-06; paired Student t-test; Figure S2c–d).

We next used a *stemness index* (*SI*) based on the *SE* to predict stemness in nine experiments of normal stem cells, including lung, muscle, prostate and iPS cells, that were not included in the compendium (Table S3). The *SI* takes two query lists as input and measures the degree to which the first list has a higher SE than the second list (see Methods). A confidence measure was also computed as the total overlap to any S-MAP module. Amazingly, in spite of low total overlap for three datasets (grey zone, Figure 6B), all normal mouse populations designated as stem cell-like obtained a higher stemness score than differentiation score (*P<*0.002, binomial test), and four of the datasets scored at least two standard deviations above randomly simulated datasets (Figure 6B).

### Cell populations enriched for cancer stem cells have higher stemness indices

Recently, several studies have linked metastasis and stem cells through self-renewal (25–27). We tested five populations from cancer studies, where cancer cells had been separated into putative cancer stem cell (CSC) and non-stem-cell populations, and two metastatic cancer populations. Intriguingly, both of the metastatic populations and three out of five of the cancer stem cell populations were classified as more stem cell-like than the corresponding normal or non-metastatic tissue (Figure 6C). To further test this, we mapped human metastatic cancer data onto the mouse genome and assigned stemness scores. Five out of fourteen datasets had too little overlap to be scored with confidence (grey zone, Figure 6D). Remarkably, all but one of the remaining datasets was classified as stem cell-like (Figure 6D). These findings strongly concur with the hypothesis that metastatic populations in both mouse and humans exhibit the molecular properties of stem cells, perhaps following an epithelial-to-mesenchymal transition. Collectively, our results demonstrate that the *SI* can be used to correctly classify both mouse and human cell populations as stem cell-like in a normal, cancer, and metastatic context.

## Discussion

The inclusion of evolutionarily related genes as modules revealed a striking number of families with expression patterns associated with stemness. It is plausible that various stem cells employ common mechanisms through the use of evolutionarily related families of proteins. Duplication and subsequent modification of genes and their enhancers through evolution may have provided the control logic needed to diversify stem cells for populating new tissue systems distinct from ancestral counterparts. Indeed, the homolog modules consistently received higher S-MAP scores compared to the modules in a previous study that did not consider gene homology [6] (Figures S15, S16).

The identification of stemness modules has obvious implications for both iPS cells and cancer therapeutics. Reprogramming candidates essential for stem cell properties are likely found among the stemness-on modules while stemness-off modules likely contain genes that have to be silent in stem cells. The SI scoring strategy, possibly adapted specifically to ESC/iPS cell signatures, could be used in the characterization of putative iPS lines. Similar strategies can be used to identify and target CSC within a heterogeneous cancer population and serve as valuable tools for fighting cancer progression. In addition, the results can be used to test for novel stem cell marker genes to improve the definition and isolation of stem cells.

The S-MAP approach synergizes with large-scale efforts to systematically extract differentially expressed genes in many different conditions represented in current microarray gene expression repositories, such as Oncomine [26] and GeneChaser [27]. It is complementary to previous approaches to uncover the mechanisms of pluri- and multi-potency maintenance, such as the development of the PluriNet [5].

In this study, we focused our analysis on modules with expression patterns ranging across a broad array of different stem cell types. However, S-MAP also revealed 152 homolog and 32 functional modules that were restricted to specific stem cell types (Table S2); these modules should reveal tissue- or lineage-specific stem cell mechanisms. The method synergizes with large-scale efforts to systematically extract differentially expressed genes in many different conditions represented in current microarray gene expression repositories, such as Oncomine [26] and GeneChaser [27]. Finally, S-MAP can be used to test for inter-species stem cell expression conservation, to characterize cancers from their normal counterparts, and is readily transferrable to other systems and diverse types of data.

## Materials and Methods

S-MAP calculates scores for gene modules, sets of related genes, based on the pattern of differential expression of each module’s constituent genes observed in several studies. It uses the scores to classify modules according to whether the genes are associated with up-regulation in stem cells (stemness-on), down-regulated in stem cells (stemness-off) or neither. S-MAP can then use the lists of stemness-related modules to classify additional datasets according to their stemness signatures using a Stemness Index score. We describe the definition of S-MAP’s non-redundant set of modules, collection of the stem cell expression compendium, and the various scores for classifying modules and datasets.

### Definition of gene modules

A non-redundant set of gene modules was collected that included both putative homolog families determined by BLAST similarity analysis and genes that participate in common pathways (Figure S1a–b). Our method used sets of functionally- and evolutionarily-related genes to determine if each set has an expression pattern significantly associated with stemness. We collected a large number of gene modules including putative homolog families and genes that participate in common pathways.

#### Homolog modules

We identified putative homolog modules by determining collections of genes sharing high protein sequence similarity. BLASTP (at an E-value cutoff <0.05) was used to align the entire mouse proteome (UCSC version mm9) containing 45,480 peptides with an associated EntrezGene identifier. For each pair of proteins, only the alignment with the highest BLASTP E-value, as well as the highest overall sequence coverage, was chosen as representative of the gene pair. Only gene pairs whose sequences had an E-value smaller than 10^−70^ and coverage of more than 50% were connected. A depth-first traversal on the resulting protein-protein similarity network was used to identify all connected components totaling 14,941 in total. Of these, 11,920 had only a single gene in the group. To incorporate more evolutionarily distant relationships and connect genes in singleton groups to more distantly related homologs, unconnected genes were assigned to the module containing the gene with the most similar protein sequence if its best match exceeded a less stringent cutoff of 10^−10^ and coverage of at least 50%. The process was iteratively repeated until singleton modules could no longer be assigned to larger gene sets. The process converged on a final set 4,659 homolog modules and decreased the number of genes remaining unconnected to 5,249. Of the homolog modules, six represented very large families of over 100 gene members. We found that the homolog module definitions were highly concordant with HomoloGene [8] (Figure S1b) even though HomoloGene was not designed specifically to delineate paralogous groups.

#### Functional modules

We collected functional modules from five different data sources including Gene Ontology [9], BioCarta’s curated pathway sets [11], CORUM’s experimentally derived mouse protein complexes [28], and putative protein complexes identified from dense sub-networks derived from BioGRID’s database of interacting proteins [29]. For BioGRID, modules were also identified from human datasets and the genes were mapped to mouse using best-reciprocal BLASTP hit analysis.

#### Non-redundant collection of modules

While the S-MAP procedure could score all collected modules, we defined a non-redundant set of modules to use for assessing the overall significance and distribution of scores. Allowing redundancy could skew the results because of highly characterized pathways. For example, Gene Ontology contains a deep hierarchy with many sub- and sub-sub-categorizations under the cell cycle process because it has been studied extensively in yeast. A redundancy filter on the functional modules helped us avoid redundant annotations of highly characterized pathways and protein complexes from dominating the results.

To filter out redundant functional modules, we first sorted the functional modules from smallest to largest and excluded any having a 25% or greater gene overlap with a smaller functional module. Functional modules with over half of the genes (50% overlap) belonging to the same set of homologs were also excluded because any such module also reflects an evolutionary connection between the genes to a greater degree than the functional connection. The relatively high overlap cutoff (50%) between evolutionary and functional gene modules allows genes with multiple functional roles to be captured by different module types. We collected all homolog and functional modules into the set *Ω*.

### Collection of a compendium of differentially expressed gene lists

We collected lists of differentially expressed genes from 30 transcriptional profiling studies, corresponding to 49 different cell populations and 12 different stem cell types. For each stem cell population in each study, we collected the list of clones found to be up-regulated relative to differentiated cells. Likewise, a differentiated-cell gene list (DGL) was constructed by including genes found to have higher expression in differentiated cells compared to stem cells. The lists were obtained or inferred either directly from the publication or by communication with the authors. To obtain a stem cell gene list (SGL), clones were mapped to their corresponding EntrezGene identifiers. If a gene had even a single matching differentially expressed clone it was included in the SGL. Clones without a corresponding EntrezGene identifier were excluded from further analysis.

For each module, a stem-cell *event matrix S* was constructed from the set of all SGLs such that *S_ij_* is empty if gene *i* was not tested in study *j*, 1 if it was found to be differentially up-regulated, and 0 otherwise. In addition to the lists representing genes over-expressed in stem cells, we also derived lists with genes over-expressed in differentiated cells (or under-expressed in stem cells). Differentiated-cell gene lists (DGLs) were obtained in an analogous fashion using the clones reported to be downregulated in each study. A DGL was obtained for all but four of the studies in the compendium. Each module was also associated with a corresponding differentiation event matrix *D* recording the up-regulation events across the DGL compendium for that module. The descriptions of the publications, cell types, and gene list sizes are listed in Dataset S1.

The recurrence score used for S-MAP (described subsequently) was motivated by the need to compensate for potential biases in combining heterogeneous results. There are obvious variations in the overlap and sizes of the SGLs and DGLs, which reflect the differences in the compared biological specimens, cell, and RNA isolation techniques, hybridization protocols, and statistical methods applied to identify differentially expressed clones. The recurrence score down-weights the influence of studies reporting highly similar gene lists to avoid a bias due to over-representation of any one stem cell type. Before computing recurrence scores for genes or modules, we calculated the similarity of all gene lists to derive groups of gene lists. Gene lists in the same group could then be collectively down-weighted in the recurrence scoring. The identification of these gene list groups is described next.

### Identifying groups of similar gene lists

Groups of similar gene lists from possibly different studies were identified by performing an all-against-all comparison of the collected gene lists, constructing a network from the pair-wise overlaps, and then unifying lists that fell into the same sub-network (Figure S1c). Gene list groups were determined by clustering similar gene lists on a network. All pairs of gene lists were compared and the significance of their overlap was determined by calculating the hypergeometric *P* value reflecting the probability of obtaining *k* overlapping genes by drawing two clusters of size *n* and *m* randomly from a genome of size *N*. Gene lists with overlap *P* values smaller than 10^−50^ were linked together to form a network of gene lists. Less stringent cutoffs did not separate gene lists from the same or different stem cell types.

Cytoscape [30] was used to ordinate and visualize the resulting network using the negative logarithm of the hypergeometric *P* values as the weights for spring-embedded layout (Figure 4). The final groups were determined by visual inspection of the network. Subnets of mutually similar gene lists of the same stem cell type were identified with the same group. This yielded three groups of sizes 2, 5, and 7 with the remaining left as degenerate groups of size 1. While the Ramalho-Santos HSC gene list [4] had overlaps with many other HSC-derived gene lists and appeared at first to warrant inclusion in the HSC group of size 5, the significance of the overlaps were borderline and less than all of the other HSC overlaps. For this reason, we left the Ramalho-Santos HSC gene list in its own degenerate group. We denoted the set of gene list groups including degenerates as *B_1_, B_2_,…, B_F_*, where *F* is the number of groups identified by the above procedure. The *B_f_*'s define a mutually exclusive and exhaustive partition of the experiments in the compendium. We write experiment *j*'s replicate group with the notation *b(j)*. For example *b(j)* = 1 is interpreted to mean the *jth* experiment was placed in the first group, *B_1_*. Thus, the notation *B_b(j)_* represents the set of gene lists grouped with a particular gene list *j* including gene list *j* itself.

### Recurrence

A score called *recurrence* was developed to measure the degree to which observed differential expression in a module is replicated across independent gene lists. Two recurrence scores were computed – one measuring repeated up-regulation across the SGLs and the other down-regulation across the DGLs. The DGL recurrence is interpreted as a measure of specificity for a module when considering its association to stemness. The recurrence score uses a meta-analysis that computes a weighted count of the number of upregulated genes across all of the studies, observed for a particular module. The score incorporates a weight for each experiment that is inversely proportionally to the number of genes upregulated in the study. Thus, studies with smaller, more specific gene lists are given higher weight than studies with large, less specific lists. The score also incorporates the redundancy of the gene lists by allowing each group of similar lists to have a single vote no matter how many lists are in a group.

A module *M* from *Ω* was considered to have a stemness-associated pattern of expression if some of its genes were found in a high proportion of SGLs. We developed a score called *recurrence* that measures the degree to which observed differential expression in a module is replicated in independent gene lists. In practice, we compute two recurrence scores – one measuring repeated up-regulation across the SGLs and the other down-regulation across the DGLs – but here we describe the calculations only for SGLs as the others for the DGLs are analogous. The recurrence score used for module *M* computed over the SGL matrix *S* is:
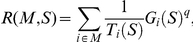
where *G_i_(S)* is a gene-specific score for gene *i* measuring its degree of up-regulatation across all of the studies, *T_i_(S)* is a normalization constant to compensate for the number of genes in the module, and the *q* exponent determines how much weight frequently expressed genes have on the score relative to less frequently expressed genes.

A gene-specific score was used that reflects the frequency with which a gene is up-regulated in the SGL compendium. Because the SGLs are of highly different sizes, we introduced a “signal strength” *z_j_* for each gene list *j* to account for the differences in specificities inherent in the experimental results. We computed the gene specific score:
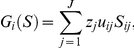
where *J* is the total number of SGLs, *u_ij_* is the unit of weight contributed by gene list *j* in its gene list group, and *S_ij_* encodes the binary event that gene *i* is upregulated in SGL *j* as defined above. The *u_ij_* term forces each gene list to contribute the same amount of weight as any other experiment in its group and is a function of the gene because different genes may be tested in different sets of experiments in the group. This term is defined as:
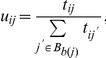
where *t_ij_* is 1 if the *i* ^th^ gene was tested in experiment *j* and 0 otherwise. Experiments in large groups get a smaller weight while experiments in small or singleton groups get the largest weight. For example, suppose three gene lists are grouped together and one gene is tested in two of the lists, while a second gene is tested in all three. For the first gene, two experiments would contribute a weight of 1/2 while, for the second gene, all experiments would contribute a weight of 1/3.

Finally, the normalization constant for the recurrence score ensures that the *z_j_u_ij_* products sum to unity to normalize for the overall size of the module; i.e. *T_i_(S)  =  Σ_j_ z_j_u_ij_*, where *j* ranges from *1* to *J*. A completely analogous score was used to compute the recurrence of a module’s down-regulated events across the compendium. Only, in this case, the *D* matrix was used in place of *S* so that each module also had an associated *R(M,D)* recurrence measure as well.

The recurrence score is similar to vote-counting meta-analysis methods that count the number of occurrences of a particular observation in a set of independent tests. In addition, as described next, it incorporates a weight for each experiment similar to the inverse variance weighting used for combining effect sizes (or inverse document frequency weighting used in information retrieval applications).

### Simulation to determine recurrence parameters

Due to the lack of appropriate positive controls, we used synthetic data to assess the accuracy of recurrence scores with different parameter choices (*q* and *z_j_*) at identifying significant up-regulation across many stem cell types.

First, the size (*n*) of each module was randomly sampled from an exponential distribution with a mean number of four genes to match the size distribution of modules in our collection. Stemness-related modules were simulated by randomly sampling three different idealized stemness patterns: 1) Many genes expressed in Many tissues (MM), 2) Single gene expressed in Many tissues (SM), and 3) Single gene expressed in a Single tissue (SS).

For each experiment, MM modules were simulated by independently drawing two genes at random such that all genes were equally likely to be selected. SM modules were simulated by first selecting a random gene from the module to have a high probability (*p* = 0.8) and the rest a low probability (p = 0.2/(n-1)). For each of the nine synthetic experiments, a single gene was chosen as up-regulated according to these probabilities. Finally, SS modules were simulated by independently choosing a single gene to be up-regulated in each experiment where each gene was equally likely to be selected.

In addition to the simulated stemness-related modules, two types of non-stemness modules were simulated: 1) tissue-specific (TS) modules in which genes were primarily expressed in a single tissue, and 2) non-related (NR) modules in which genes exhibited up-regulated events expected by chance. First, a set of preferentially up-regulated genes *U* was simulated by selecting each gene independently in the module with probability *p = *0.5. For TS modules, a random experiment was selected and assigned a higher probability (*p = 0.8*) relative to the other experiments (*p = 0.2/(J-1))*. TS modules were then simulated by allowing only genes in *U* to be up-regulated, each up-regulated in one tissue chosen according to these biased probabilities. For NR modules, the simulation was similar but all experiments had an equal probability of selection.

Nine experiments were simulated for 2000 modules among which 120 represented stemness modules (MM, SM, and SS in equal proportions). The remaining non-related modules were simulated in the ratio 2:3 (TS:NR).

For the *q* exponent, we tested values of 0.5, 1, 2, and 3. For the signal strength *z_j_*, three different functions of the size of gene list *j* were tested – *1/v_j_*, *(1-v_j_)*, *-log(v_j_)* – where *v_j_* corresponds to the size of gene list *j.* For *q = 2*, we also tested an additional *z_j_* assignment, *z_j_* = 1, equivalent to the use of no weighting.

All 13 combinations were evaluated for their ability to find different stemness-associated patterns in synthetic data, and the results were tabulated in Table S2. The area-under-the-curve (AUC) was calculated for the different *q* and *z_j_* choices. To estimate the AUC, we ranked all simulated stemness-related and unrelated modules by their scores. Precision and recall were computed by sweeping through cutoffs in the recurrence score. False positives were assumed to be those unrelated modules with scores above the cutoff; false-negatives were assumed to be those related modules with scores below the cutoff.

The critical parameter in the simulation was *q*. Smaller values of *q* produced more accurate results for modules that use multiple genes in multiple tissues, while higher values of *q* produced more accurate results for modules that use a single gene in many tissues. The combination *q = 2* and *z_j__ =  -log(v_j_)* gave the highest average AUC among all combinations (Table S2) and was used for the analysis.

### Diversity scoring

A *cell-diversity* score based on information theory was used to distinguish between modules that are up-regulated in many, compared to few, stem cell types. The score is computed on the relative proportions of up-regulated genes in the module rather than on the absolute proportions. Thus, even a module expressing a small number of genes on average can have a high diversity if the same (small) fraction of its genes is expressed across many cell types. In contrast, a module with a low diversity has a higher relative proportion of up-regulated genes in one or a few stem cell types compared to the relative proportion in other types.

A relative frequency vector was derived from a module’s event matrix by computing, for each cell type *l*, the relative proportion of its genes found to be up-regulated across all SGLs of type *l*:
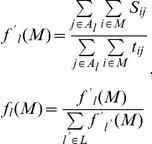
where *A_l_* represents the set of experiments of cell type *l* and *L* is the total number of such cell types. The second equation above defines *f’_l_* to be the relative frequency of the module genes present in any SGLs of type *l*. The cell-diversity of a module is defined as the normalized entropy over these relative frequencies:
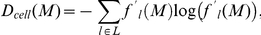
where *log(x)* is the base 2 logarithm of *x*. *D_cell_(M)* ranges between 0 and log *|L| “*bits,” with a module obtaining a value near the maximum when its up-regulated events are roughly equal across all cell types.

An analogous *gene-diversity* score was defined to quantify how evenly utilized the genes are across the studies. The gene-diversity score *D_gene_(M)* was calculated on the transpose of the event matrix. Cell- and gene-diversity scores were computed over the DGLs. We computed normalized versions of cell-diversity, denoted *D_cell_’(M)*, by dividing by *log |L|*, and of gene diversity, denoted *D_gene_’(M),* by dividing by *log |n|*, to provide an intuitive measure of the diversity ranging between 0 and 1.

### Significance of the recurrence and diversity scores

The significance of the recurrence and cell-diversity scores was determined by simulating randomly drawn modules with matched sizes to generate size-specific false discovery rates. Each module could then be compared to a thousand permuted modules of the same size to identify those with significantly scores.

The data within each experiment were permuted to produce 1,000 different SGL compendiums and their corresponding event matrices. The total number of up-regulated genes in each experiment was kept the same, but a random set of genes of the same size was simulated as up-regulated. The permutations preserved the correlation structure between gene lists in the same gene list group (Figure S1c) by keeping blocks of data values for each gene within each group together. This procedure produced randomly generated event matrices 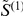, 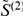, …, 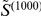 from which an empirical distribution for the recurrence was obtained by calculating the recurrence for each module and each permuted event matrix. Because we expected the distribution of the recurrence to be influenced by the size of a module, we grouped modules into six size classes 1, 2, 3, 4, 5-10, and >10 and denote the modules in the *k* ^th^ class as *Ω(k)* (Figure S2a). From this null distribution, we estimated the false-discovery rate of a recurrence score for a module of size-class *k* as:
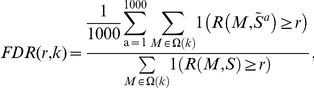
where *1(x)* is the indicator function equal to 1 if the argument *x* is true and 0 otherwise. To identify all modules with a score associated with an FDR of 5% or less, we determined a critical value *c* for each module size class that gave *FDR(c,k)*  = 0.05.

We used a similar random sampling approach to assess the significance of the cell-diversity scores. Randomized families from 1,000 permutations were used to generate cell-type diversity scores for random modules. For each module size, a false discovery rate was estimated for various cell-diversity cutoffs ranging between 0 and 3.6 bits (log (12) for the 12 stem cell types). Inspection of these cutoffs suggested that the FDR estimates for modules of different sizes were highly comparable (Figure S2c). Therefore, a single overall cell-diversity cutoff of 2.5, calculated as a weighted average across all modules, was used to achieve a desired FDR rate of 5%. Any module with a cell-diversity exceeding 2.5 was considered “cell type diverse.”

Because modules have a variety of different genes, identifying FDR cutoffs is problematic, especially for small modules. Therefore, a simple cutoff was used to distinguish modules utilizing many genes versus those utilizing a few. Modules with calculated normalized gene diversity scores higher than 0.5 were considered “gene-diverse”.

### Specificity score

While individual genes in a module could be exclusively expressed in some stem cells, if a module has many such genes (differentially expressed in a diverse set of cell types), it may not always be stemness-specific. For example, even a recurrent module could have a subset of genes switched on in stem cells and a different subset switched on in differentiated cells. To identify modules with highly specific patterns, we used the recurrence score computed on the DGLs as a measure of specificity. If a module’s SGL recurrence had an FDR less than 5% and its DGL recurrence had an FDR higher than 95%, it was classified as “highly specific”. Modules with DGL recurrence FDR between 5% and 95% were classified as “moderately specific.” Highly and moderately specific modules were included in the stemness categories.

### Pattern classification

For all modules found to have significant recurrence (FDR <5%), we classified their pattern of expression using cell- and gene-diversity. Restricting our investigation to the recurrent modules is expected to reduce false positive inferences but may miss some interesting biological phenomena. Based on the cell- and gene-diversity scores, modules were assigned to one of six possible categories: all-for-all (AFA), one-for-all (OFA), constitutive module (CM), and constitutive gene (CG), all-for-one (AFO), and one-for-one (OFO). The definitions used were as follows: AFAs, OFAs, CMs and CGs had cell-diversities of at least 2.5 bits of information entropy, while AFOs and OFOs had lower cell-diversity values. AFAs, AFOs and CMs had normalized gene-diversities of at least 0.5 while OFAs, OFOs, and CGs had lower gene-diversity values. CMs and CGs were non-specific, as they showed significant recurrence in both SGLs and DGLs and may reflect housekeeping functions. AFA and OFA modules were defined as stemness modules and are referred to as stemness-on if identified from the SGLs and stemness-off if from the DGLs.

### Deriving an expression map of stemness modules

Each module was represented as a vector of fractions of upregulated genes in each cell type. We calculated the Pearson correlation between all pairs of modules. The large number of correlations was reduced by a filtering step, such that only correlations higher than 0.8 were kept. To reduce the number of spurious unrelated connections, at least one of the two compared modules was required to be recurrent. The modules (nodes) and their correlations (edges) were loaded into Cytoscape. A force-directed layout algorithm was used to position the modules onto the X-Y plane such that the correlations were treated as pair-wise force constraints among the modules.

### Stemness index (SI)

We used the set of stemness-on modules *M^+^* and stemness-off modules *M^−^* identified by S-MAP to develop a score capable of recognizing that a new query set of differentially expressed genes *Q* is enriched for stemness modules. We defined the *Stemness Index* (*SI*) as a contrast between the overlap of the query with modules indicative of stemness compared to those indicative of differentiated cells. To this end, we defined the Stemness Overlap (SO) to be the weighted overlap of *Q* with all of the stemness modules – the higher the overlap, the more likely the query was derived from genes up-regulated in stem cells. To increase the sensitivity of the score for identifying new modules involved in core stemness, overlaps with modules of high cell diversity were more highly weighted compared to modules of lower diversity. We defined the weight of influence for a module in proportion to its cell-diversity as 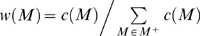. The SO could then be defined as:
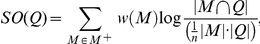
where *M* ∩ *Q* represents the observed fraction of overlap between the query *Q* and module M; the denominator in the logarithm represents the fraction of overlap expected by chance. The form of the SO score is similar to a log-likelihood ratio where each term in the summation can be thought of as the log-probability that the query would be observed given it was produced by module *M*, contrasted by the log-probability of it being produced by chance. A Differentiation Overlap (DO) was calculated identically except that *M^−^* was used in place of *M^+^* for the calculation of the module weights and the score. For a given list of genes, we compute the Stemness Enrichment (SE) as half of the difference between the SO and DO scores. A positive SE indicates that the gene list *Q* has a higher overlap with stemness modules compared to differentiated modules on average.

The stemness index (SI) score assigns a single number to a type-II study, one that compares two populations. Each type-II study has two associated gene lists: a positive gene list containing genes upregulated in the experimental population (e.g. stem cells) and a negative gene list containing genes upregulated in the control population (e.g. differentiated cells). Let *Q^+^* and *Q^−^* represent the postive and negative gene lists for an experiment respectively. An experiment that induces stemness-related modules to the exclusion of differentiation-related modules will receive a high SE(*Q^+^*). By the same token, the same experiment could also repress differentiation modules to the exclusion of stemness modules detected in the negative list and therefore receive an extremely negative SE(*Q^−^*). To capture both of these indicators of stemness, we defined the stemness index (SI) score as the difference between the SE(*Q^+^*) and the SE(*Q^−^*). A positive SI indicates that the experiment’s positive gene list has higher correspondence with stemness-on compared to stemness-off modules relative to the experiment’s own negative gene list. Values near zero indicate either that an experiment’s queries have little overlap with any stemness-on or –off modules, or that they overlap with modules from both in equal proportions. For this reason, we also consider the total overlap of these gene lists against SI. The total module overlap (*TMO*) was computed as the sum of all of the module overlap scores for both the positive and negative gene lists; i.e. *TMO(Q^+^,Q^−^)*  = 1/2 * (*SO(Q^+^) + SO(Q^−^) + DO(Q^+^) + DO(Q^−^))*.

To assess the self-consistency of the SI score, we used a 5-fold cross-validation framework. In each cross-validation run, we held out 20% of the SGLs (and their corresponding DGLs). We then identified stemness-on and –off modules using recurrence, cell-diversity, and specificity applied to the remaining 80% of the gene lists. The SI was then calculated on all of the lists in the held-out 20%.

We compared the ability of homolog versus functional modules to contribute accurate information to the *SI*. Each set was used without the other. Surprisingly, homolog modules showed higher precision than functional modules at all levels of recall (Figure S6a). The precision-recall plot showed significant levels of precision compared to a control in which *SI* was computed on random modules (Figure S6b).

### Assessing the significance of the stemness index and total module overlap

We asked whether the observed *SI* and *TMO* scores were significant. We constructed a background distribution for these scores by simulating pairs of random gene lists. For both the up-regulated and down-regulated genes in an experimental set, we simulated random gene lists of the same size by drawing without replacement from the set of all genes tested in the experiment. This sampling procedure was repeated twenty times to generate twenty pairs of matched upregulated and downregulated gene lists for each experimental data set. The *SI* and *TMO* were calculated on all simulated pairs. Because we expect the variability in the *SI* to increase with an increase in *TMO* (because, for example, extremely positive or negative SI’s must necessarily have high *TMO* levels), we estimated both the mean and standard deviation of the simulated pairs as a function of their *TMO*. We used windows of width equal to one *TMO* unit and incremented by 0.1 units.

### Detection of Enriched Biological Categories

We compiled a collection of functional categories from Gene Ontology [9], BioCarta [11], KEGG [31] and MIPS [32] into a single set of functional gene groups. To avoid any categories that may be too general, we restricted the collection to functional categories that had 100 member genes or less. For each module, we assessed the significance of its overlap with every individual functional category using the hypergeometric distribution to estimate a *P*-value (PV) as:
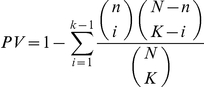
where *k* is the number of genes in the module that are also in functional group, *n* is the total number of genes in the module, *K* is the number of genes in the functional group, and *N* is the number of total genes with any functional annotation. All *P-*values were corrected for multiple hypotheses testing using the Bonferonni correction and a significance level of 0.01 was chosen for further analysis.

### Stemness Index

We developed a *Stemness Index* (*SI*) to contrast the degree to which a study upregulates modules associated with stemness compared to those associated with differentiated cells. For a particular study, we are given two lists of genes: one representing genes up-regulated (*Q^+^*) and another representing those down-regulated (*Q^−^*) in the conditions of the study. Both *Q^+^* and *Q^−^* are overlapped with every S-MAP module to compute a score reflecting the total overlap to stemness and differentiated modules, weighted by each module’s *cell-diversity*. A total overlap to all S-MAP modules provides an indication of how relevant a prediction of stemness might be to the study. For studies found to have at least some non-random level of overlap, we can then ask whether the upregulated genes show a biased correspondence to stemness or differentiated modules – i.e. *Q^+^* may overlap with stemness modules in higher proportion than *Q^−^* or vice versa. To quantify this intuition, we used a stemness enrichment (*SE*) score to measure the difference in overlap between stemness modules and differentiated modules for both *Q^+^* and *Q^−^*. The final *SI* was then defined as *SE(Q^+^)-SE(Q^−^)*.

## Supporting Information

Figure S1 **A. A Protein sequence similarity BLASTP-based approach yields homolog-based gene modules.** BLASTP is used to generate alignments between all proteins (white nodes) in the mouse proteome and at a stringent cutoff, depth-first search (DFS) is applied to identify all connected components: homolog families. Lines connecting genes indicate that the gene pair satisfies the cutoff criteria. Subsequently, an iterative neighbor expansion technique is applied to all singletons (red) until the set of homolog modules converges to its final form. **B. Overlap of homolog modules with HomoloGene indicates an 88% correspondence between our BLASTP-based homolog modules and the HomoloGene paralog groups (left).** In most cases HomoloGene groups are smaller and are identified as subsets of our BLAST-derived homolog modules (right). This is most likely because the HomoloGene groups identify only very recent paralog occurrences (most recent common ancestor at the split of rodents). The highest discrepancy between homolog group assignments comes from the assignments of putative and predicted genes with no known associated descriptions, or gene names. **C. Groups of gene lists determined from a similarity network.** Each node represents a gene list; color indicates stem cell type. Edges connect two gene lists of significant overlap, and can connect either gene lists derived from the same (red) or different (blue) stem cell types. Gene list groups used for recurrence scoring are circled in black.
(TIFF)

Figure S2 **A. A false discovery approach to recurrence identifies 266 significantly recurrent homolog modules in the compendium.** The x-axis corresponds to the size-dependent recurrence score, while the y-axis shows the false discovery rate. Each color represents the FDR curve associated with a different module size. The value under each colored arrow represents the number of up-regulated homolog families of that size that passed the recurrence cutoff for that size. FDR cutoff used to identify significantly recurrent modules was 5%. **B. Separation of stem cells into cultured and non-cultured groups detected little polarization impact.** Each circle in the Venn diagram represents the number of recurrent modules identified using each type of input data: cultured-cell-only input (pink), primary cell-only input (blue), and combined cell input (yellow). The thick black dashed line demarks the set of recurrent modules that have primary cell contribution. The moon-shaped area represents 54% of the recurrent modules identified using the whole compendium. **C**. FDR analysis to determine a significant cell-diversity cutoff. A cutoff was determined for the cell-diversity by averaging results from various module sizes (different line styles and colors). The FDR (x-axis) was plotted against a sweep of the cell-diversity cutoff (x-axis) **D**. The number of modules (y-axis) at or exceeding the cell diversity value (x-axis) aligned with the x-axis in (C). The cutoff was selected as the 5% FDR cutoff score associated with the weighted average of the FDR curves for all family sizes (A). Each color represents the FDR curve associated with a different module size. X-axis represents the cell diversity score, while the y-axis shows the FDR in log scale. To facilitate log-plotting, a floor value of 0.0001 was selected for all entries that would be otherwise 0. At the 5% FDR cutoff, 114 recurrent homolog families (red) passed the criteria and were labeled as cell-type diverse modules (lower panel). **E. Various module sizes displayed significant shifts in recurrent up-regulation scores.** Recurrence score distribution of all homolog modules of size 2 (black), compared to randomized modules of size 2, based on 1,000 random permutations of the original data. X-axis shows the recurrent up-regulation score; y-axis indicates the number of modules in each bin. **F**. Same as A, but for modules of size 4 genes. **G**. Same as A, but for modules of size 5 genes.
(TIFF)

Figure S3 **Homolog modules (black) are more enriched for modules with higher recurrence scores than modules from the Stem Cell Module Map of Wong *et al.* (2008) (green).** Histograms show the proportion of modules (*y*-axis) that had a given range of *excess recurrence* for all modules (*x*-axis). Excess recurrence was defined as a module’s recurrence score minus the recurrence corresponding to the 5% FDR cutoff for that module’s size.
(TIFF)

Figure S4 **Ingenuity Pathway Analysis of stemness modules.** Ingenuity Pathway Analysis identified two networks associated with stemness-on component B (A-B), two networks associated with stemness-on component C (C-D), and a network associated with stemness-off modules (E).
(TIFF)

Figure S5 Each module is represented by two heatmaps (delineated by each dotted rectangle) – the upper heatmap represents the expression of every gene in the module in each stem cell type and can range from 0 (no sGLs of a given stem cell type express highly a given gene) to 1 (all sGLs of a given stem cell type express highly a given gene). The lower heatmap in each case shows the average up-regulation state of every gene in the module in every differentiated cell type. Gray represents missing data, or the inability to calculate an average because of missing data in either the stem cell or differentiated cell experiments. Abbreviations: HSC – hematopoietic stem cells, ESC – embryonic; NSC – neural; MaSC – mammary; MSC – mesenchymal; LiSC – liver; InSC – intestinal; RPC – retinal; GEP – gastric; TSC – trophoblast; SSC – spermatogonial; HBSC - hair bulge (epithelial) stem cells. **A. Chromatin-associated modules were highly represented among the stemness-on modules. B. Wnt signaling-associated modules were well represented among the stemness-on modules.** At least six different Wnt-related modules are scored by S-MAP as stem-cell specific – Sfrp, Tcf, Tle, Fzd, alpha-catenin, and delta-catenin. **C. DNA-repair-associated modules were highly represented among the stemness-on modules.** Seven different repair-related modules are scored by S-MAP as stem-cell specific –Msh, Exo1, Rad51-related, p53, Terf, Brca1, and Pcna. **D. Several important transcriptional regulator modules were well represented among the stemness-on modules – Myc, Myb, Pbx, and Id (Inhibitor of Differentiation). E. Cell cycle-related and DNA replication-associated modules were also well represented among the stemness-on modules by several modules – Mcm, Cdt1, Pcna, Orc1 and Cdc6.** Most genes represented in these modules are so frequently expressed that they score as stemness genes in S-MAP on their own.
(TIFF)

Figure S6 **A. Precision-recall comparison of twelve stemness index (SI) scores, based on homolog-only (red), functional-only (blue) and combined (green) features shows a superior performance of the homolog-based predictors over the functional feature-based predictors.** X-axis measures the recall associated with each method, while the y-axis measure the precision of each method. The most accurate method should be approximately in the top right hand-side corner. The comparison between the twelve stemness index scoring measures suggests that the multiplicative-based log-ratio method, based on a homolog-based feature set (red dashed line) has the highest accuracy. **B. Precision-recall comparison of the real stemness and differentiation features to 100 randomly selected feature sets.** The red line indicates the performance of the real feature set of stemness and differentiation homolog modules, while the black dashed line shows the average performance of 100 random homolog feature sets of the same size as the original feature set. The real stemness and differentiation features perform significantly better than the average random feature sets.
(TIFF)

Dataset S1 Lists of all homolog and functional modules tested with associated S-MAP scores, their corresponding stem cell recurrence scores, stem cell diversity scores, differentiated cell recurrence scores, differentiated cell diversity scores, and their stem cell and differentiated cell gene diversity scores. Each score can also have a ‘hi’, ‘mod’, and ‘lo’ status assigned. Stemness-on/off status, as well as module type classification (if available) is provided, where appropriate. All functional modules available have been scored and shown, but only the non-redundant subset (611 modules; labeled as “non-redundant” in the table) is discussed in this paper.
(XLS)

Dataset S2 Stem cell compendium gene lists for all studies examined by S-MAP. Each worksheet represents an individual population gene list with annotation of the study name, stem cell type, SGL/DGL membership, EntrezGene, and gene name annotation.
(XLS)

Dataset S3 List of all 40 stemness genes identified by S-MAP, along with their corresponding stem cell recurrence and diversity scores. Gene names, annotations, and descriptions are provided for all genes.
(XLS)

Table S1 Summary of the studies in the stem cell compendium.
(DOC)

Table S2 Area under the curve (AUC) for 13 different recurrence parameter combinations.
(DOC)

Table S3 List of independent studies tested with the SI score.
(DOC)
