# Supplementary figures and images for: Computational Integration of Homolog and Pathway Gene Module Expression Reveals General Stemness Signatures
Source: PLoS One. 2011 Apr 29;6(4):e18968. doi: 10.1371/journal.pone.0018968 (PMC3084730; doi:10.1371/journal.pone.0018968)

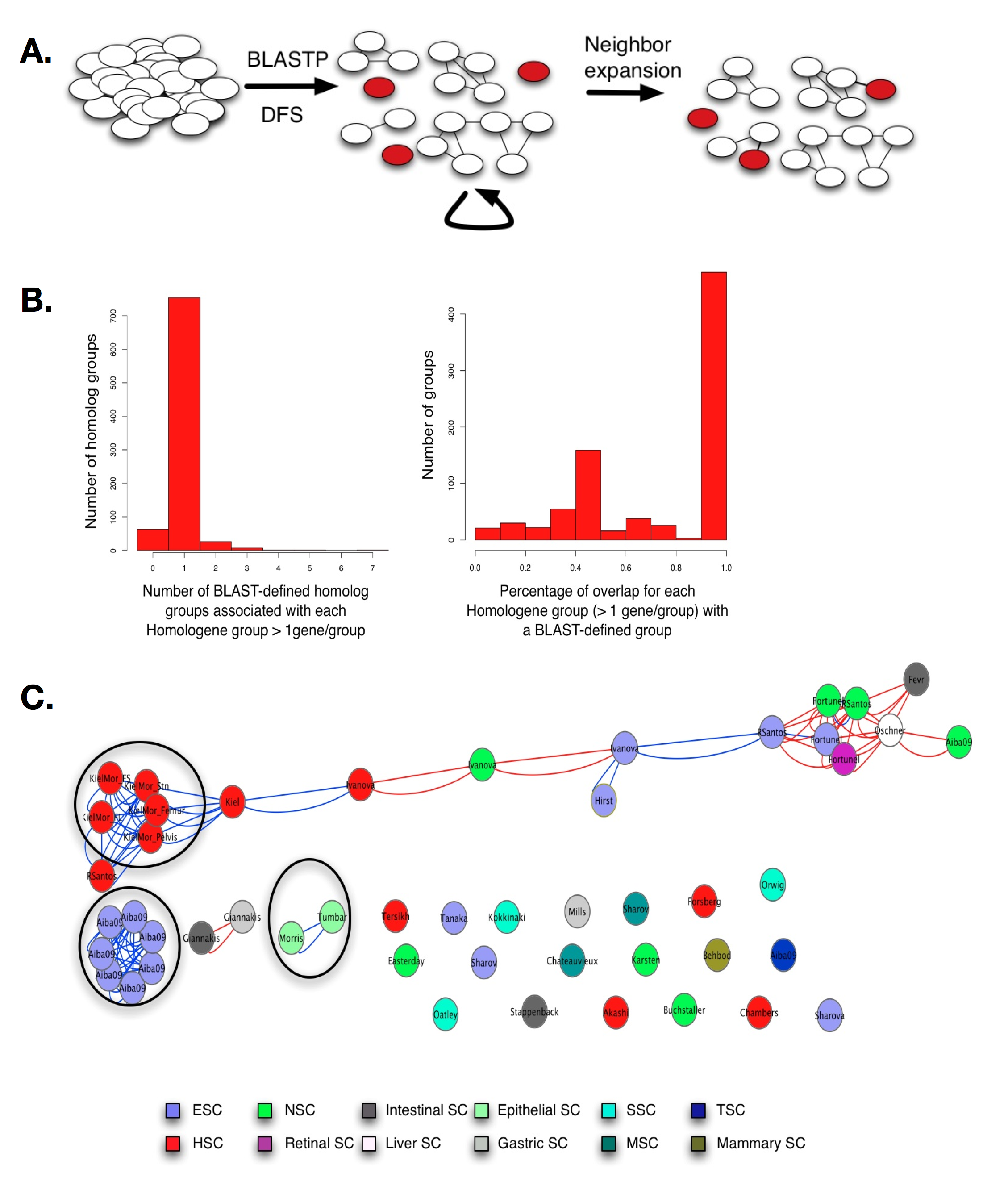

Supplement: Figure S1 — A. A Protein sequence similarity BLASTP-based approach yields homolog-based gene modules. BLASTP is used to generate alignments between all proteins (white nodes) in the mouse proteome and at a stringent cutoff, depth-first search (DFS) is applied to identify all connected components: homolog families. Lines connecting genes indicate that the gene pair satisfies the cutoff criteria. Subsequently, an iterative neighbor expansion technique is applied to all singletons (red) until the set of homolog modules converges to its final form. B. Overlap of homolog modules with HomoloGene indicates an 88% correspondence between our BLASTP-based homolog modules and the HomoloGene paralog groups (left). In most cases HomoloGene groups are smaller and are identified as subsets of our BLAST-derived homolog modules (right). This is most likely because the HomoloGene groups identify only very recent paralog occurrences (most recent common ancestor at the split of rodents). The highest discrepancy between homolog group assignments comes from the assignments of putative and predicted genes with no known associated descriptions, or gene names. C. Groups of gene lists determined from a similarity network. Each node represents a gene list; color indicates stem cell type. Edges connect two gene lists of significant overlap, and can connect either gene lists derived from the same (red) or different (blue) stem cell types. Gene list groups used for recurrence scoring are circled in black. (TIFF) [file pone.0018968.s001.tif]

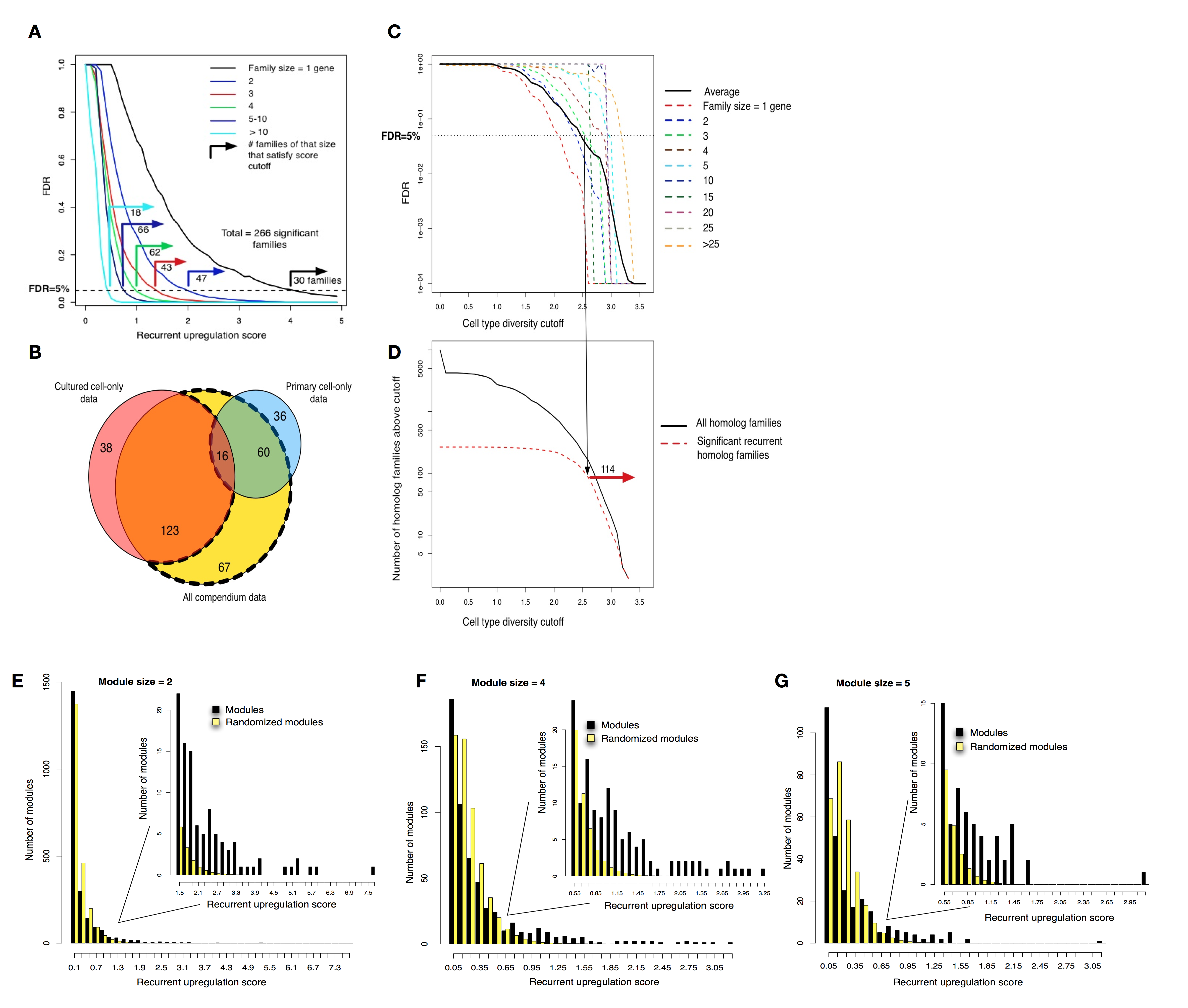

Supplement: Figure S2 — A. A false discovery approach to recurrence identifies 266 significantly recurrent homolog modules in the compendium. The x-axis corresponds to the size-dependent recurrence score, while the y-axis shows the false discovery rate. Each color represents the FDR curve associated with a different module size. The value under each colored arrow represents the number of up-regulated homolog families of that size that passed the recurrence cutoff for that size. FDR cutoff used to identify significantly recurrent modules was 5%. B. Separation of stem cells into cultured and non-cultured groups detected little polarization impact. Each circle in the Venn diagram represents the number of recurrent modules identified using each type of input data: cultured-cell-only input (pink), primary cell-only input (blue), and combined cell input (yellow). The thick black dashed line demarks the set of recurrent modules that have primary cell contribution. The moon-shaped area represents 54% of the recurrent modules identified using the whole compendium. C. FDR analysis to determine a significant cell-diversity cutoff. A cutoff was determined for the cell-diversity by averaging results from various module sizes (different line styles and colors). The FDR (x-axis) was plotted against a sweep of the cell-diversity cutoff (x-axis) D. The number of modules (y-axis) at or exceeding the cell diversity value (x-axis) aligned with the x-axis in (C). The cutoff was selected as the 5% FDR cutoff score associated with the weighted average of the FDR curves for all family sizes (A). Each color represents the FDR curve associated with a different module size. X-axis represents the cell diversity score, while the y-axis shows the FDR in log scale. To facilitate log-plotting, a floor value of 0.0001 was selected for all entries that would be otherwise 0. At the 5% FDR cutoff, 114 recurrent homolog families (red) passed the criteria and were labeled as cell-type diverse modules (lower panel). E. Various [file pone.0018968.s002.tif]

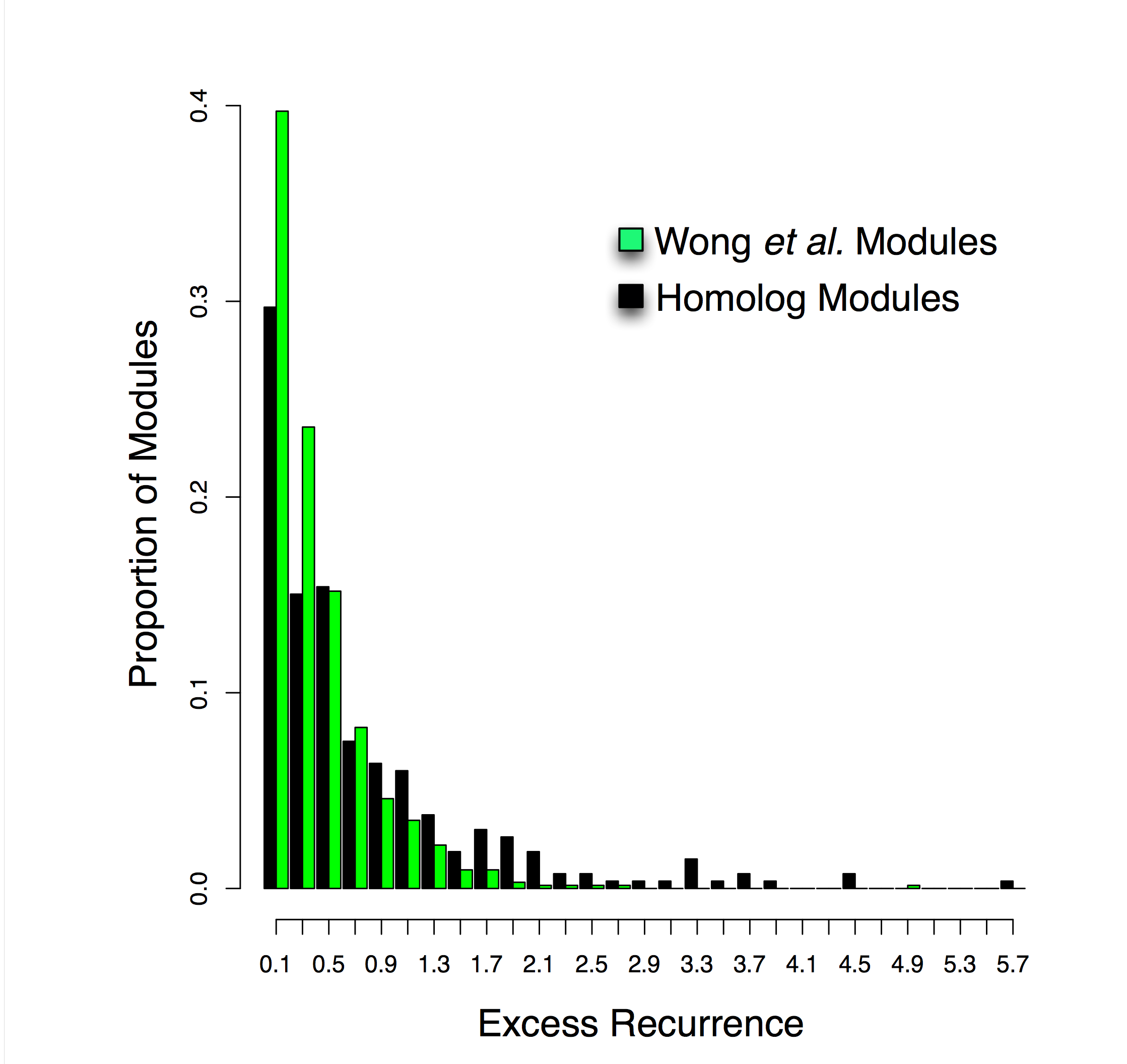

Supplement: Figure S3 — Homolog modules (black) are more enriched for modules with higher recurrence scores than modules from the Stem Cell Module Map of Wong et al. (2008) (green). Histograms show the proportion of modules (y-axis) that had a given range of excess recurrence for all modules (x-axis). Excess recurrence was defined as a module’s recurrence score minus the recurrence corresponding to the 5% FDR cutoff for that module’s size. (TIFF) [file pone.0018968.s003.tif]

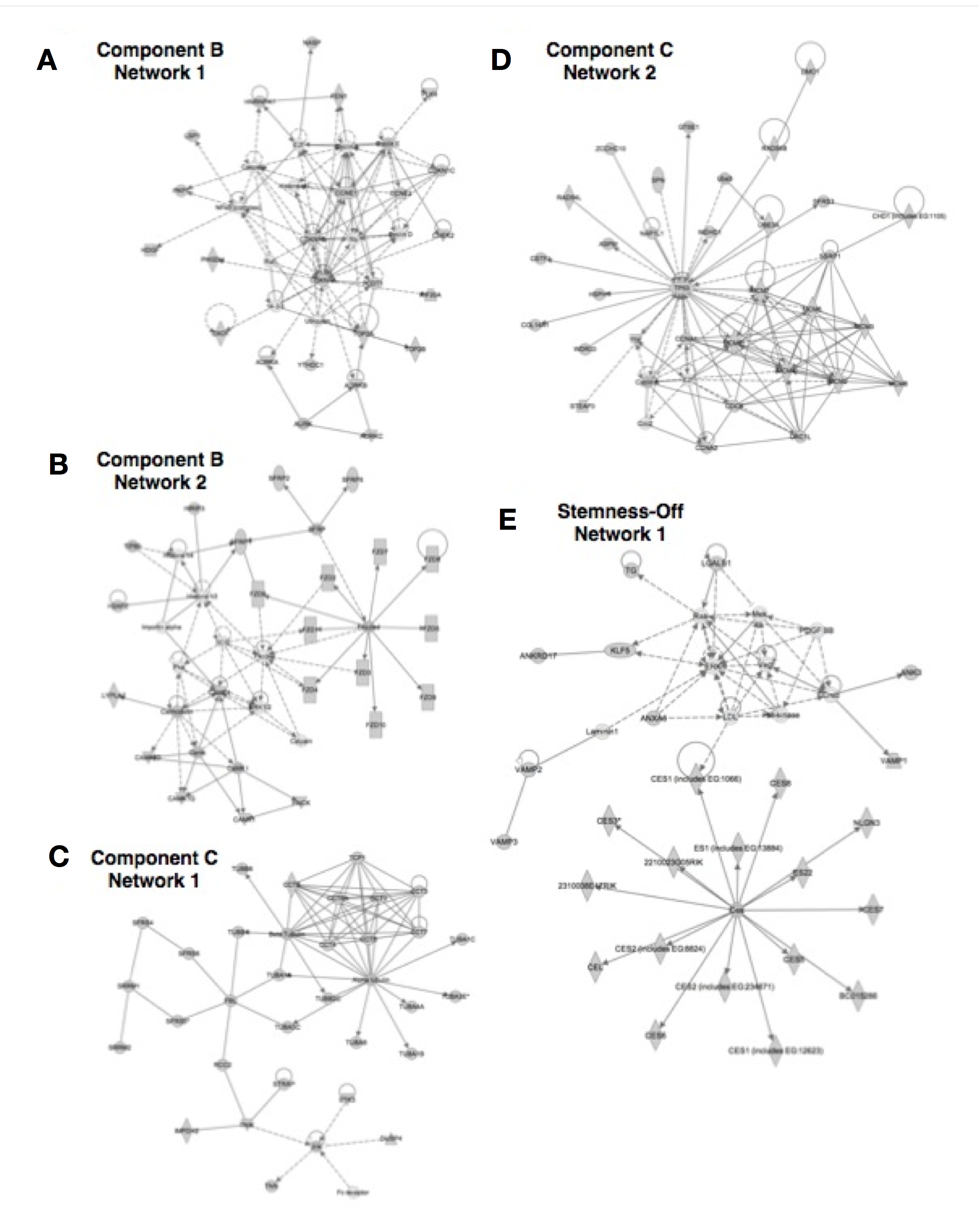

Supplement: Figure S4 — Ingenuity Pathway Analysis of stemness modules. Ingenuity Pathway Analysis identified two networks associated with stemness-on component B (A-B), two networks associated with stemness-on component C (C-D), and a network associated with stemness-off modules (E). (TIFF) [file pone.0018968.s004.tif]

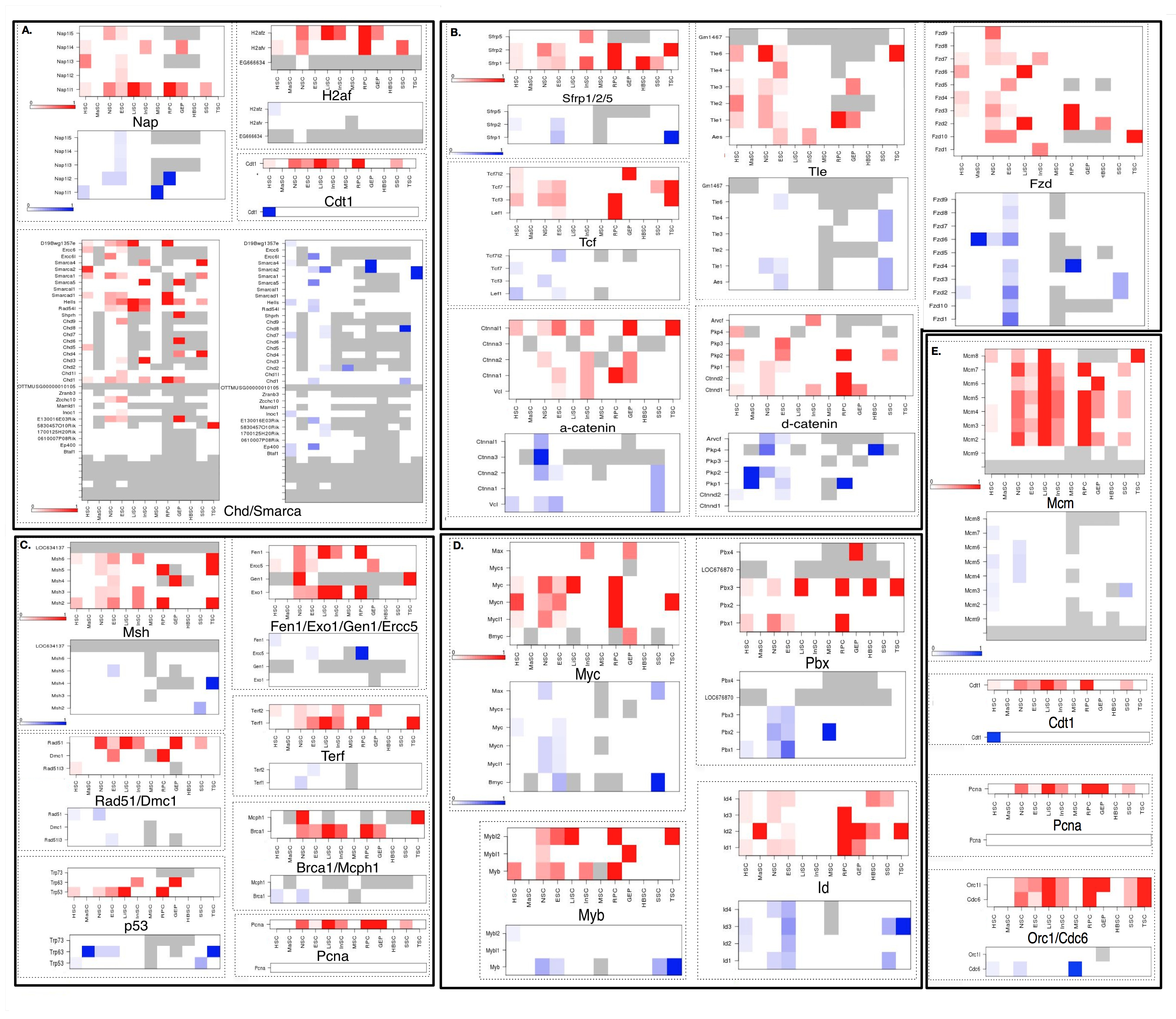

Supplement: Figure S5 — Each module is represented by two heatmaps (delineated by each dotted rectangle) – the upper heatmap represents the expression of every gene in the module in each stem cell type and can range from 0 (no sGLs of a given stem cell type express highly a given gene) to 1 (all sGLs of a given stem cell type express highly a given gene). The lower heatmap in each case shows the average up-regulation state of every gene in the module in every differentiated cell type. Gray represents missing data, or the inability to calculate an average because of missing data in either the stem cell or differentiated cell experiments. Abbreviations: HSC – hematopoietic stem cells, ESC – embryonic; NSC – neural; MaSC – mammary; MSC – mesenchymal; LiSC – liver; InSC – intestinal; RPC – retinal; GEP – gastric; TSC – trophoblast; SSC – spermatogonial; HBSC - hair bulge (epithelial) stem cells. A. Chromatin-associated modules were highly represented among the stemness-on modules. B. Wnt signaling-associated modules were well represented among the stemness-on modules. At least six different Wnt-related modules are scored by S-MAP as stem-cell specific – Sfrp, Tcf, Tle, Fzd, alpha-catenin, and delta-catenin. C. DNA-repair-associated modules were highly represented among the stemness-on modules. Seven different repair-related modules are scored by S-MAP as stem-cell specific –Msh, Exo1, Rad51-related, p53, Terf, Brca1, and Pcna. D. Several important transcriptional regulator modules were well represented among the stemness-on modules – Myc, Myb, Pbx, and Id (Inhibitor of Differentiation). E. Cell cycle-related and DNA replication-associated modules were also well represented among the stemness-on modules by several modules – Mcm, Cdt1, Pcna, Orc1 and Cdc6. Most genes represented in these modules are so frequently expressed that they score as stemness genes in S-MAP on their own. (TIFF) [file pone.0018968.s005.tif]

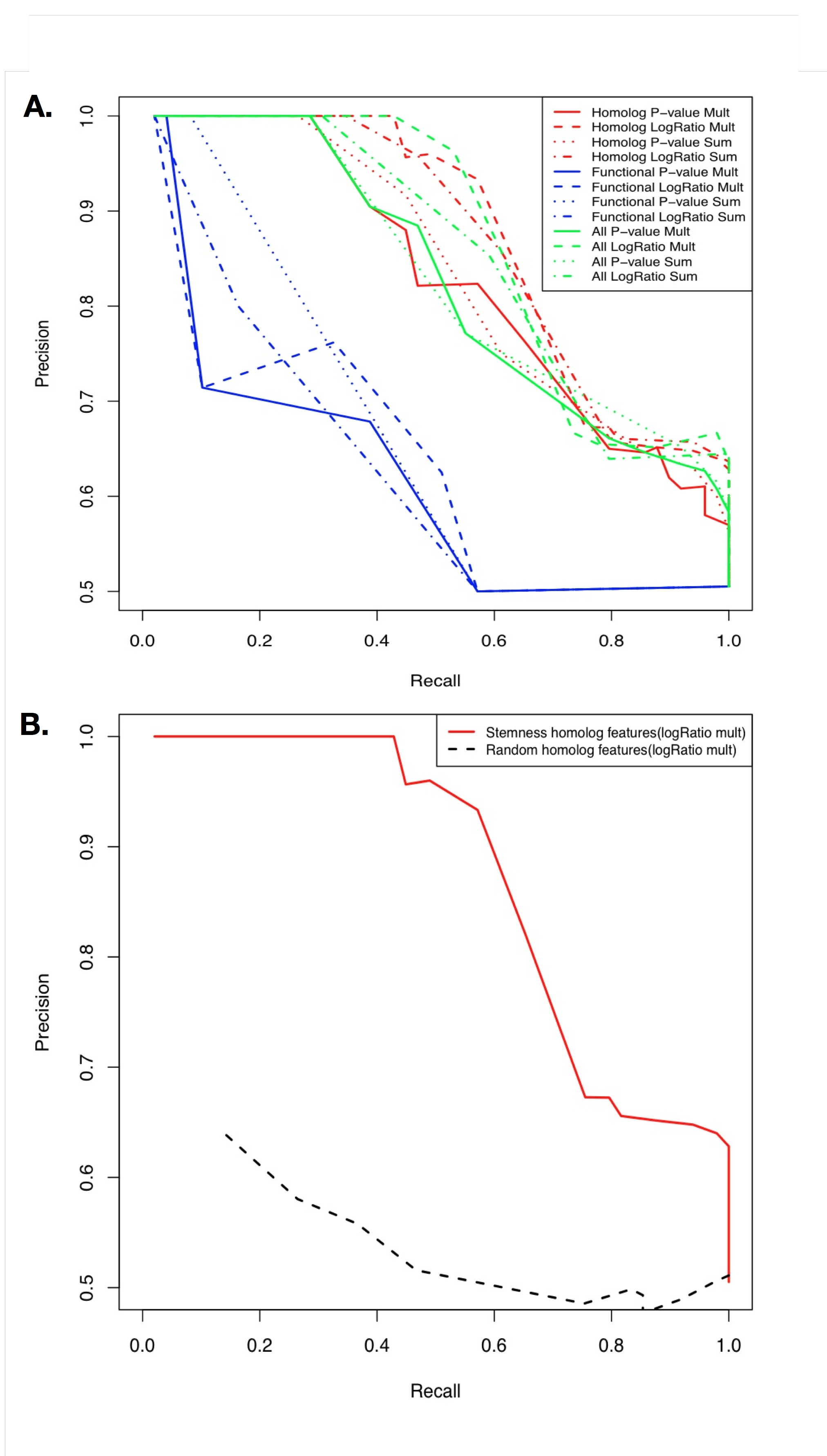

Supplement: Figure S6 — A. Precision-recall comparison of twelve stemness index (SI) scores, based on homolog-only (red), functional-only (blue) and combined (green) features shows a superior performance of the homolog-based predictors over the functional feature-based predictors. X-axis measures the recall associated with each method, while the y-axis measure the precision of each method. The most accurate method should be approximately in the top right hand-side corner. The comparison between the twelve stemness index scoring measures suggests that the multiplicative-based log-ratio method, based on a homolog-based feature set (red dashed line) has the highest accuracy. B. Precision-recall comparison of the real stemness and differentiation features to 100 randomly selected feature sets. The red line indicates the performance of the real feature set of stemness and differentiation homolog modules, while the black dashed line shows the average performance of 100 random homolog feature sets of the same size as the original feature set. The real stemness and differentiation features perform significantly better than the average random feature sets. (TIFF) [file pone.0018968.s006.tif]
