# Supplementary material for: Computational Integration of Homolog and Pathway Gene Module Expression Reveals General Stemness Signatures
Source: PLoS One. 2011 Apr 29;6(4):e18968. doi: 10.1371/journal.pone.0018968 (PMC3084730; doi:10.1371/journal.pone.0018968)
Supplement: Table S1 — Summary of the studies in the stem cell compendium. (DOC) [file pone.0018968.s010.doc]

Table S1. Summary of the studies in the stem cell compendium.

| Publication | Stem cell population (label) | Primary or cultured | SGL size | Differentiated cell population | dGL size |
| --- | --- | --- | --- | --- | --- |
| Ivanova, *et al.* [1] | HSC (A)^a^ | Primary | 701 | MBC | 457 |
|  | ESC (B) | Cultured | 577 | MBC | 1842 |
|  | NSC (C) | Cultured | 636 | MBC | 2155 |
| Ramalho-Santos, *et al.* [2] | HSC (A) | Primary | 1272 | BM main population | 750 |
|  | ESC (B) | Cultured | 1185 | BM main pop.+ lat.ventricle | 229 |
|  | NSC (C) | Cultured | 1585 | Lateral ventricle | 884 |
| Fortunel, et al [3] | ESC (B) | Cultured | 1118 | PEF (primary embryonic fibroblasts) | 3981 |
|  | NSC (C) | Cultured | 1150 | Lateral ventricle | 1164 |
|  | RPC (D) | Primary | 1487 | Mature retinas | 1358 |
| Forsberg, et al [4] | LT-HSC (A) | Primary | 398 | MPP | 260 |
| Akashi, et al [5] | LT-HSC (A) | Primary | 701 | CLP | 376 |
|  |  |  |  | CMP | 581 |
| Kiel, et al. [6] | FL-HSC (A) | Primary | 307 | FL-CD45+ | 154 |
|  | FS-HSC (A) | Primary | 211 | FL-CD45+ | 117 |
|  | Femur-HSC (A) | Primary | 353 | Femur-CD45+ | 329 |
|  | Pelvis-HSC (A) | Primary | 262 | Femur-CD45+ | 308 |
|  | Sternum-HSC (A) | Primary | 273 | Femur-CD45+ | 322 |
| Kiel et al. [7] | HSC (A) | Primary | 917 | CD45+ cells | 625 |
| Terskikh, et al. [8] | HSC (A) | Primary | 30 | CMP | 5 |
|  |  |  |  | GMP | 9 |
|  |  |  |  | MEP | 8 |
|  |  |  |  | ProB | 9 |
|  |  |  |  | ProT | 15 |
| Buchstaller, et al. [9] | NCSC (C) | Cultured | 189 | Schwann cells | 257 |
| Chambers, et al. [10] | HSC (A) | Primary | 272 | Various downstream cell types (intersection) | 23 |
| Chateauvieux, et al [11] | MSC (E) | Cultured | 254 | Non-MSC (2018; BFC012) lines | 172 |
| Easterday, et al [12] | NS (C) (neurosphere cultures | Cultured | 65 | DC (differentiated cultures) | 13 |
| Hirst, et al. [13] | ESC (B) | Cultured | 461 | EB (d4) | n/a |
| Karsten, et al. [14] | NSC (C) | Cultured | 117 | 24hDC | 48 |
| Mills, et al. [15] | GEP (F) | Primary | 135 | Parietal cells | 107 |
| Stappenback, et al. [16] | SiEP (G) | Primary | 158 | Paneth cell- normal crypt base epithelium | 1 |
| Oschner, et al. [17] | BMEL (basal) (H) | Cultured | 716 | BMEL (d5 aggregate) | 483 |
| Behbod, et al. [18] | MG-SP (I) | Cultured | 269 (75)^b^ | MG-NSP | 90 (111)^a^ |
| Sharova, et al. [19] | ESC/EGC (B) | Cultured | 1462 | DC (LIF) | 441 |
|  |  |  |  | DC(+RA) | 1566 |
| Morris, et al. [20] | Hair follicle SC (J) | Primary | 75 | Keratinocytes | 49 |
| Tumbar, et al. [21] | LRC (J) | Primary | 106 | Keratinocyte progeny | n/a |
| Tanaka, et al. [22] | ES/TS (B) | Cultured | 23 | MEF cells | 48 |
| Fevr, et al. [23] | Intestinal SC (G) | Primary | 427 | -beta-catenin (withdrawal) | 541 |
| Aiba, et al. [24] | ESC (B) | Cultured | 632 | F lineage | 1261 |
|  | ESC (B) | Cultured | 1782 | G lineage | 794 |
|  | ESC (B) | Cultured | 1464 | N lineage | 1429 |
|  | ESC (B) | Cultured | 1695 | Z lineage | 1847 |
|  | ESC (B) | Cultured | 1108 | P0/P4 | 1111 |
|  | iPS (B) | Cultured | 1739 | MEF | 2250 |
|  | iPS (B) | Cultured | 1765 | MEF | 2198 |
|  | NSC (C) | Cultured | 799 | DC | 1128 |
|  | TSC (K) | Cultured | 1207 | Placenta | 2118 |
| Oatley, et al. [25] | SSC (L) | Cultured | 176 | -GDNF (withdrawal) | 59 |
| Orwig, et al. [26] | SSC (L) | Primary | 319 | Testicular somatic cells | 1769 |
| Kokkinaki, et al. [27] | SSC (L) | Primary | 276 | Spermatogonia | 103 |
| Sharov, et al. [28] | ES/EG/TS (B) | Cultured | 97 | n/a | n/a |
|  | MS/NS/HS (E) | Cultured | 24 | n/a | n/a |
| Giannakis, et al [29] | GEP (F) | Primary | 1365 | n/a | n/a |
|  | SiEP (G) | Primary | 1440 | n/a | n/a |

^a^ Letters indicate the stem cell type: A, hematopoietic stem cells; B, embryonic stem cells; C, neural stem cells; D; retinal stem cells; E, mesenchymal stem cells; F, gastric stem cells; G, intestinal stem cells; H, liver stem cells; I, mammary stem cells; J, epithelial stem cells; K, trophoblast stem cells; and L, spermatogonial stem cells.

^b^ Due to an error in data processing, SGLs and dGLs for mammary cells contained mixtures of up- and down-regulated genes that were used for recurrence scoring. The corrected sizes are shown in brackets; all heatmaps online and in the manuscript were also updated to include the corrected data.

**REFERENCES**

1. Ivanova NB, Dimos JT, Schaniel C, Hackney JA, Moore KA, et al. (2002) A stem cell molecular signature. Science 298: 601--604.

2. Ramalho-Santos M, Yoon S, Matsuzaki Y, Mulligan RC, Melton DA (2002) "Stemness": transcriptional profiling of embryonic and adult stem cells. Science 298: 597--600.

3. Fortunel NO, Otu HH, Ng H-H, Chen J, Mu X, et al. (2003) Comment on " 'Stemness': transcriptional profiling of embryonic and adult stem cells" and "a stem cell molecular signature". Science 302: 393; author reply 393.

4. Forsberg EC, Prohaska SS, Katzman S, Heffner GC, Stuart JM, et al. (2005) Differential expression of novel potential regulators in hematopoietic stem cells. PLoS Genet 1: e28.

5. Akashi K, He X, Chen J, Iwasaki H, Niu C, et al. (2003) Transcriptional accessibility for genes of multiple tissues and hematopoietic lineages is hierarchically controlled during early hematopoiesis. Blood 101: 383--389.

6. Kiel MJ, Yilmaz OH, Iwashita T, Yilmaz OH, Terhorst C, et al. (2005) SLAM family receptors distinguish hematopoietic stem and progenitor cells and reveal endothelial niches for stem cells. Cell 121: 1109--1121.

7. Kiel MJ, Iwashita T, Yilmaz OH, Morrison SJ (2005) Spatial differences in hematopoiesis but not in stem cells indicate a lack of regional patterning in definitive hematopoietic stem cells. Dev Biol 283: 29--39.

8. Terskikh AV, Miyamoto T, Chang C, Diatchenko L, Weissman IL (2003) Gene expression analysis of purified hematopoietic stem cells and committed progenitors. Blood 102: 94--101.

9. Buchstaller J, Sommer L, Bodmer M, Hoffmann R, Suter U, et al. (2004) Efficient isolation and gene expression profiling of small numbers of neural crest stem cells and developing Schwann cells. J Neurosci 24: 2357--2365.

10. Chambers SM, Boles NC, Lin K-YK, Tierney MP, Bowman TV, et al. (2007) Hematopoietic fingerprints: an expression database of stem cells and their progeny. Cell Stem Cell 1: 578--591.

11. Chateauvieux S, Ichante J-L, Delorme B, Frouin V, Pietu G, et al. (2007) Molecular profile of mouse stromal mesenchymal stem cells. Physiol Genomics 29: 128--138.

12. Easterday MC, Dougherty JD, Jackson RL, Ou J, Nakano I, et al. (2003) Neural progenitor genes. Germinal zone expression and analysis of genetic overlap in stem cell populations. Dev Biol 264: 309--322.

13. Hirst CE, Ng ES, Azzola L, Voss AK, Thomas T, et al. (2006) Transcriptional profiling of mouse and human ES cells identifies SLAIN1, a novel stem cell gene. Dev Biol 293: 90--103.

14. Karsten SL, Kudo LC, Jackson R, Sabatti C, Kornblum HI, et al. (2003) Global analysis of gene expression in neural progenitors reveals specific cell-cycle, signaling, and metabolic networks. Dev Biol 261: 165--182.

15. Mills JC, Andersson N, Hong CV, Stappenbeck TS, Gordon JI (2002) Molecular characterization of mouse gastric epithelial progenitor cells. Proc Natl Acad Sci U S A 99: 14819--14824.

16. Stappenbeck TS, Mills JC, Gordon JI (2003) Molecular features of adult mouse small intestinal epithelial progenitors. Proc Natl Acad Sci U S A 100: 1004--1009.

17. Ochsner SA, Strick-Marchand H, Qiu Q, Venable S, Dean A, et al. (2007) Transcriptional profiling of bipotential embryonic liver cells to identify liver progenitor cell surface markers. Stem Cells 25: 2476--2487.

18. Behbod F, Xian W, Shaw CA, Hilsenbeck SG, Tsimelzon A, et al. (2006) Transcriptional profiling of mammary gland side population cells. Stem Cells 24: 1065--1074.

19. Sharova LV, Sharov AA, Piao Y, Shaik N, Sullivan T, et al. (2007) Global gene expression profiling reveals similarities and differences among mouse pluripotent stem cells of different origins and strains. Dev Biol 307: 446--459.

20. Morris RJ, Liu Y, Marles L, Yang Z, Trempus C, et al. (2004) Capturing and profiling adult hair follicle stem cells. Nat Biotechnol 22: 411--417.

21. Tumbar T, Guasch G, Greco V, Blanpain C, Lowry WE, et al. (2004) Defining the epithelial stem cell niche in skin. Science 303: 359--363.

22. Tanaka TS, Kunath T, Kimber WL, Jaradat SA, Stagg CA, et al. (2002) Gene expression profiling of embryo-derived stem cells reveals candidate genes associated with pluripotency and lineage specificity. Genome Res 12: 1921--1928.

23. Fevr T, Robine S, Louvard D, Huelsken J (2007) Wnt/beta-catenin is essential for intestinal homeostasis and maintenance of intestinal stem cells. Mol Cell Biol 27: 7551--7559.

24. Aiba K, Nedorezov T, Piao Y, Nishiyama A, Matoba R, et al. (2009) Defining developmental potency and cell lineage trajectories by expression profiling of differentiating mouse embryonic stem cells. DNA Res 16: 73--80.

25. Oatley JM, Avarbock MR, Telaranta AI, Fearon DT, Brinster RL (2006) Identifying genes important for spermatogonial stem cell self-renewal and survival. Proc Natl Acad Sci U S A 103: 9524--9529.

26. Orwig KE, Ryu B-Y, Master SR, Phillips BT, Mack M, et al. (2008) Genes involved in post-transcriptional regulation are overrepresented in stem/progenitor spermatogonia of cryptorchid mouse testes. Stem Cells 26: 927--938.

27. Kokkinaki M, Lee T-L, He Z, Jiang J, Golestaneh N, et al. (2009) The molecular signature of spermatogonial stem/progenitor cells in the 6-day-old mouse testis. Biol Reprod 80: 707--717.

28. Sharov AA, Piao Y, Matoba R, Dudekula DB, Qian Y, et al. (2003) Transcriptome analysis of mouse stem cells and early embryos. PLoS Biol 1: E74.

29. Giannakis M, Stappenbeck TS, Mills JC, Leip DG, Lovett M, et al. (2006) Molecular properties of adult mouse gastric and intestinal epithelial progenitors in their niches. J Biol Chem 281: 11292--11300.
