# Supplementary material for: Computational Integration of Homolog and Pathway Gene Module Expression Reveals General Stemness Signatures
Source: PLoS One. 2011 Apr 29;6(4):e18968. doi: 10.1371/journal.pone.0018968 (PMC3084730; doi:10.1371/journal.pone.0018968)
Supplement: Table S2 — Area under the curve (AUC) for 13 different recurrence parameter combinations. (DOC) [file pone.0018968.s011.doc]

**Table S2.** **Area under the curve (AUC) for 13 different recurrence parameter combinations.**

| Scoring method^a^ | Type SM AUC^b^ | Type SS AUC^c^ | Type MM AUC^d^ | Average AUC^e^ |
| --- | --- | --- | --- | --- |
| q=0.5; z_j_=-log(v_j_) | 0.3087 | 0.5036 | *0.6565* | 0.4896 |
| q=0.5; z_j_=1/v_j_ | 0.3076 | 0.5010 | 0.6556 | 0.4880 |
| q=0.5; z_j_=1-v_j_ | 0.3086 | 0.5035 | *0.6565* | 0.4895 |
| q=1; z_j_=-log(v_j_) | 0.3844 | *0.5137* | 0.6481 | 0.5154 |
| q=1; z_j_=1/v_j_ | 0.3844 | *0.5137* | 0.6481 | 0.5153 |
| q=1; z_j_=1-v_j_ | 0.3843 | *0.5137* | 0.6481 | 0.5153 |
| **q=2; z_j_=-log(v_j_)^f^** | **0.4733** | **0.4867** | **0.6147** | **0.5249** |
| q=2; z_j_=1/v_j_ | 0.4731 | 0.4862 | 0.6152 | 0.5248 |
| q=2; z_j_=1-v_j_ | 0.4731 | 0.4868 | 0.6148 | 0.5249 |
| q=3; z_j_=-log(v_j_) | 0.5005 | 0.4781 | 0.5952 | 0.5246 |
| q=3; z_j_=1/v_j_ | *0.5013* | 0.4774 | 0.5949 | 0.5246 |
| q=3; z_j_=1-v_j_ | 0.5009 | 0.4781 | 0.5951 | 0.5246 |
| q=2; no weight | 0.4727 | 0.4840 | 0.6147 | 0.5238 |

**^a^** Parameter combination used in the recurrence score.

^b^ AUC results for modules with expression of a single gene in many tissues.

^c^ AUC results for modules with expression of a single gene in a single tissue.

^d^ AUC results for modules with expression of multiple genes in multiple tissues.

^e^ Average AUC across all module types.

^f^ Parameter combination chosen for analysis in the main text.
