# Supplementary material for: Computational Integration of Homolog and Pathway Gene Module Expression Reveals General Stemness Signatures
Source: PLoS One. 2011 Apr 29;6(4):e18968. doi: 10.1371/journal.pone.0018968 (PMC3084730; doi:10.1371/journal.pone.0018968)
Supplement: Table S3 — List of independent studies tested with the SI score. (DOC) [file pone.0018968.s012.doc]

**Table S3: List of independent studies tested with the SI score.**

|  |  | **No.** |
| --- | --- | --- |
| **Author, Year^a^** | **Type of cells** | **pop.^b^** |
| Blum R, 2009 [1] | Normal | 3 |
| Meadows E, 2008 [2] | Normal | 2 |
| Hu G, 2009 [3] | Normal | 1 |
| Pedemonte E, 2007 [4] | Normal | 1 |
| Rock J, 2009 [5] | Normal | 2 |
| Stingl J, 2006 [6] | Normal | 2 |
| Kendrick H, 2008 [7] | Normal | 2 |
| Senju S., 2009 [8] | Normal | 2 |
| Rochon C, 2006 [9] | Side populations | 2 |
| Gulati A, 2008 [10] | Side populations | 2 |
| Liadaki K, 2005 [11] | Side populations | 4 |
| Zhang M, 2008 [12] | CSC | 2 |
| Somervaille T, 2009 [13] | CSC | 2 |
| Cho R, 2008 [14] | CSC | 2 |
| Krivtsov AV, 2006 [15] | CSC | 2 |
| Read TA, 2009 [16] | CSC | 2 |
| Williams C, 2009 [17] | CSC | 2 |
| Crawford N, 2008 [18] | Metastatic | 2 |
| Gibbons D, 2009 [19] | Metastatic | 2 |

**^a^** First author (and year) of the study from which gene lists were taken.

^b^ Number of tested populations.

**REFERENCES**

1. Blum R, Gupta R, Burger PE, Ontiveros CS, Salm SN, et al. (2009) Molecular signatures of prostate stem cells reveal novel signaling pathways and provide insights into prostate cancer. PLoS One 4: e5722.

2. Meadows E, Cho J-H, Flynn JM, Klein WH (2008) Myogenin regulates a distinct genetic program in adult muscle stem cells. Dev Biol 322: 406--414.

3. Hu G, Kim J, Xu Q, Leng Y, Orkin SH, et al. (2009) A genome-wide RNAi screen identifies a new transcriptional module required for self-renewal. Genes Dev 23: 837--848.

4. Pedemonte E, Benvenuto F, Casazza S, Mancardi G, Oksenberg JR, et al. (2007) The molecular signature of therapeutic mesenchymal stem cells exposes the architecture of the hematopoietic stem cell niche synapse. BMC Genomics 8: 65.

5. Rock JR, Onaitis MW, Rawlins EL, Lu Y, Clark CP, et al. (2009) Basal cells as stem cells of the mouse trachea and human airway epithelium. Proc Natl Acad Sci U S A 106: 12771--12775.

6. Stingl J, Eirew P, Ricketson I, Shackleton M, Vaillant F, et al. (2006) Purification and unique properties of mammary epithelial stem cells. Nature 439: 993-997.

7. Kendrick H, Regan JL, Magnay FA, Grigoriadis A, Mitsopoulos C, et al. (2008) Transcriptome analysis of mammary epithelial subpopulations identifies novel determinants of lineage commitment and cell fate. BMC Genomics 9: 591.

8. Senju S, Haruta M, Matsunaga Y, Fukushima S, Ikeda T, et al. (2009) Characterization of dendritic cells and macrophages generated by directed differentiation from mouse induced pluripotent stem cells. Stem Cells 27: 1021--1031.

9. Rochon C, Frouin V, Bortoli S, Giraud-Triboult K, Duverger V, et al. (2006) Comparison of gene expression pattern in SP cell populations from four tissues to define common "stemness functions". Exp Cell Res 312: 2074--2082.

10. Gulati AS, Ochsner SA, Henning SJ (2008) Molecular properties of side population-sorted cells from mouse small intestine. Am J Physiol Gastrointest Liver Physiol 294: G286-294.

11. Liadaki K, Kho AT, Sanoudou D, Schienda J, Flint A, et al. (2005) Side population cells isolated from different tissues share transcriptome signatures and express tissue-specific markers. Exp Cell Res 303: 360--374.

12. Zhang M, Behbod F, Atkinson RL, Landis MD, Kittrell F, et al. (2008) Identification of tumor-initiating cells in a p53-null mouse model of breast cancer. Cancer Res 68: 4674--4682.

13. Somervaille TCP, Matheny CJ, Spencer GJ, Iwasaki M, Rinn JL, et al. (2009) Hierarchical maintenance of MLL myeloid leukemia stem cells employs a transcriptional program shared with embryonic rather than adult stem cells. Cell Stem Cell 4: 129--140.

14. Cho RW, Wang X, Diehn M, Shedden K, Chen GY, et al. (2008) Isolation and molecular characterization of cancer stem cells in MMTV-Wnt-1 murine breast tumors. Stem Cells 26: 364-371.

15. Krivtsov AV, Twomey D, Feng Z, Stubbs MC, Wang Y, et al. (2006) Transformation from committed progenitor to leukaemia stem cell initiated by MLL-AF9. Nature 442: 818--822.

16. Read T-A, Fogarty MP, Markant SL, McLendon RE, Wei Z, et al. (2009) Identification of CD15 as a marker for tumor-propagating cells in a mouse model of medulloblastoma. Cancer Cell 15: 135--147.

17. Williams C, Helguero L, Edvardsson K, Haldosen LA, Gustafsson JA (2009) Gene expression in murine mammary epithelial stem cell-like cells shows similarities to human breast cancer gene expression. Breast Cancer Res 11: R26.

18. Crawford NP, Alsarraj J, Lukes L, Walker RC, Officewala JS, et al. (2008) Bromodomain 4 activation predicts breast cancer survival. Proc Natl Acad Sci U S A 105: 6380-6385.

19. Gibbons DL, Lin W, Creighton CJ, Zheng S, Berel D, et al. (2009) Expression signatures of metastatic capacity in a genetic mouse model of lung adenocarcinoma. PLoS One 4: e5401.
